# Location dependent coordination chemistry and MRI relaxivity, in *de novo* designed lanthanide coiled coils†
†Electronic supplementary information (ESI) available: Methods, peptide characterization data including mass spectrometry and analytical HPLC, sedimentation equilibrium data, circular dichroism, luminescence, and NMR data. See DOI: 10.1039/c5sc04101e


**DOI:** 10.1039/c5sc04101e

**Published:** 2015-12-22

**Authors:** Matthew R. Berwick, Louise N. Slope, Caitlin F. Smith, Siobhan M. King, Sarah L. Newton, Richard B. Gillis, Gary G. Adams, Arthur J. Rowe, Stephen E. Harding, Melanie M. Britton, Anna F. A. Peacock

**Affiliations:** a School of Chemistry , University of Birmingham , Edgbaston , B15 2TT , UK . Email: a.f.a.peacock@bham.ac.uk; b National Centre for Macromolecular Hydrodynamics , School of Biosciences , University of Nottingham , Sutton Bonington , LE12 5RD , UK; c School of Health Sciences , The University of Nottingham , Queen's Medical Centre , Nottingham , NG7 2HA , UK

## Abstract

Herein, we establish for the first time the design principles for lanthanide coordination within coiled coils, and the important consequences of binding site translation. By interrogating design requirements and by systematically translating binding site residues, one can influence coiled coil stability and more importantly, the lanthanide coordination chemistry. A 10 Å binding site translation along a coiled coil, transforms a coordinatively saturated Tb(Asp)_3_(Asn)_3_ site into one in which three exogenous water molecules are coordinated, and in which the Asn layer is no longer essential for binding, Tb(Asp)_3_(H_2_O)_3_. This has a profound impact on the relaxivity of the analogous Gd(iii) coiled coil, with more than a four-fold increase in the transverse relaxivity (21 to 89 mM^–1^ s^–1^), by bringing into play, in addition to the outer sphere mechanism present for all Gd(iii) coiled coils, an inner sphere mechanism. Not only do these findings warrant further investigation for possible exploitation as MRI contrast agents, but understanding the impact of binding site translation on coordination chemistry has important repercussions for metal binding site design, taking us an important step closer to the predictable and truly *de novo* design of metal binding sites, for new functional applications.

## Abstract 2

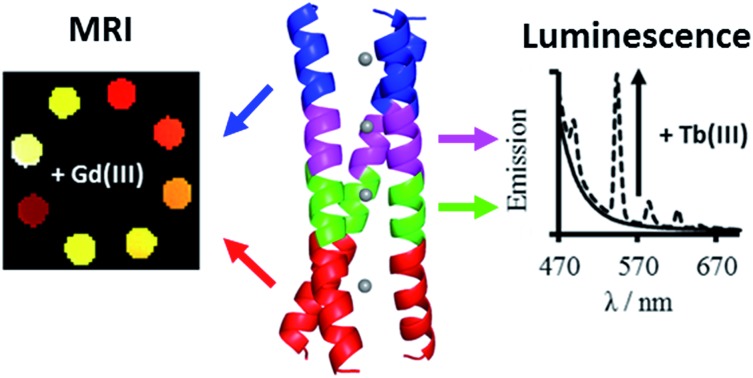
Lanthanide binding site translation linearly along a coiled coil has a large impact on stability, coordination chemistry, and MRI relaxivity.

## Introduction

Metal ions are essential in many biological processes, with an estimated one third of all proteins requiring a metal ion to function.^1^ However, despite the extensive use of metal ions in biology, nature selects from a rather limited range. This likely reflects their “bio-availability”, with metal ions buried deep in the earth's crust being unlikely candidates, but it does allow one to contemplate, what type of chemistry might the inclusion of non-biological or *xeno* metal ions, allow biology to achieve.

Not surprisingly, a number of examples exist in which either the native metal ion is replaced with a *xeno* metal, or alternatively, a new site is specifically engineered into a biomolecule for subsequent *xeno* metal binding. Notable examples of relevance to this work involve the introduction of lanthanide metal ions, with no known biological role, for their attractive magnetic and photophysical properties. For example, lanthanide-binding tags (LBTs) have been developed, commonly inspired by native calcium binding sites, for introduction into protein sequences. Their high affinity for lanthanide ions allows for their use as luminescent probes to solve protein dynamics, structural restraints and distancing in NMR, as well as potential applications as MRI contrast agents.^2^–^5^

Rather than changing the metal, there has been much interest in replacing native protein ligands with miniature artificial protein scaffolds, often designed *de novo* (from first-principles). The fact that they represent much simpler systems with which to establish important structure–function relationships, make them attractive for exploitation. The large majority of *de novo* peptides used for metal ion coordination, have focused on the coiled coil, a supercoil of α-helices, which can be designed predictably.^6^–^9^ Notable achievements include the successful reproduction of biologically relevant mononuclear sites, such as the active site of carbonic anhydrase,^10^ dinuclear complexes, including dioxygen-activating di-iron sites,^11^ multinuclear clusters, *e.g.* the cubane-like [4Fe–4S] cluster,^12^ and introduction of inorganic cofactors such as the dioxygen binding heme.^13^ Importantly, these metallocoiled coils can be used to address key questions about native sites, and fundamental questions about metalloprotein coordination chemistry. For example, Pecoraro and co-workers demonstrated that the maximal rate, solvent/substrate access and metal binding affinity of the ZnHis_3_O carbonic anhydrase mimetic site, are dependent on its location within the coiled coil.^14^

The large majority of *de novo* metallocoiled coil examples have focused their efforts on mimicking the active sites of native metalloproteins, *vide supra*. However, our approach is to combine both strategies and to develop artificial proteins complexed to *xeno* metals, with the view to developing new functional systems for valuable applications beyond what can be currently offered by Nature (*e.g.* MRI contrast agents). Only a few examples like these exist in the literature, and include a designed three-stranded helical bundle capable of sequestering uranyl (UO_2_^2+^) from seawater;^15^ as well as several short reports of lanthanide coiled coils.^16^–^18^ We recently reported the first ever gadolinium coiled coil, interrogated its coordination chemistry, and demonstrated, despite the lack of any inner sphere water, its promising magnetic resonance contrast capabilities.^19^

**Fig. 1:**
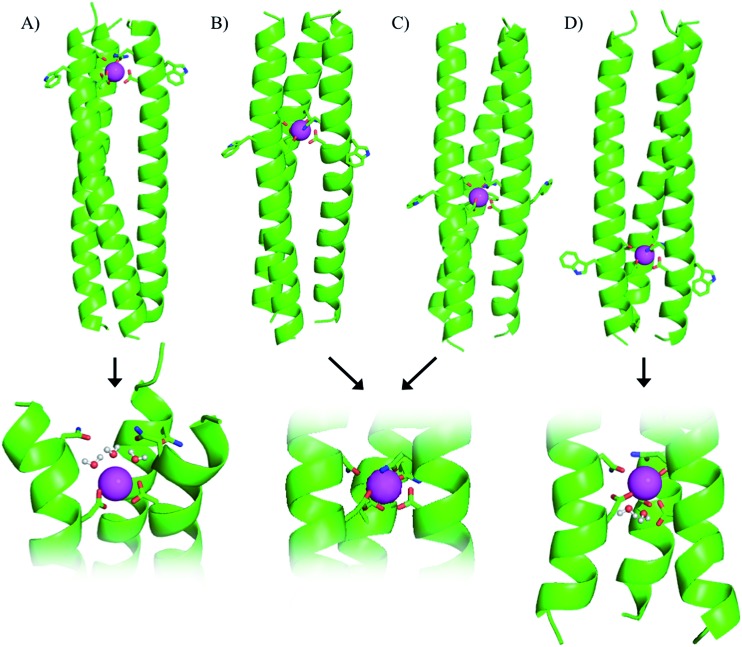
Cartoon representation showing side-on views of (A) Ln(MB1-1)_3_, (B) Ln(MB1-2)_3_, (C) Ln(MB1-3)_3_ and (D) Ln(MB1-4)_3_. Proposed models of binding sites, including bound water molecules, shown at the bottom of the figure. Shown are the main chain atoms represented as helical ribbons (green), the Asn, Asp and Trp side chains in stick form (oxygen in red and nitrogen in blue), and the Ln(iii) ion as a sphere (pink).

Our gadolinium binding site was engineered within the hydrophobic core of a parallel three stranded coiled coil, based on five I_a_A_b_A_c_I_d_E_e_Q_f_K_g_ heptad repeats, by introducing an aspartic acid (Asp, D) at an *a* site and an asparagine (Asn, N) in the *d* site located directly above, so as to provide up to nine O-donor ligands for lanthanide coordination. A tryptophan was introduced adjacent to the binding site (at a *f* position), as its ability to sensitize terbium emission allowed us to monitor and probe terbium coordination directly. The resulting peptide, MB1-2 (see Fig. 1B and Table 1), was found to fold in the presence of trivalent lanthanide ions and bind coordinatively saturated terbium. Despite the lack of any coordinated water, often an important feature of gadolinium MRI contrast agents, the Gd(MB1-2)_3_ complex displayed superior MRI relaxivity (efficiency) when compared to Dotarem®, a small molecule gadolinium complex currently used in the clinic.^19^

**Table 1:** Peptide sequences used in this study

Peptide	Sequence
MB1-1	Ac-G IAA**N**E**W**K **D**AAIEQK IAAIEQK IAAIEQK IAAIEQK G-NH_2_
MB1-2	Ac-G IAAIEQK IAA**N**E**W**K **D**AAIEQK IAAIEQK IAAIEQK G-NH_2_
MB1-3	Ac-G IAAIEQK IAAIEQK IAA**N**E**W**K **D**AAIEQK IAAIEQK G-NH_2_
MB1-4	Ac-G IAAIEQK IAAIEQK IAAIEQK IAA**N**E**W**K **D**AAIEQK G-NH_2_
CS1-1	Ac-G IAAIE**W**K **D**AAIEQK IAAIEQK IAAIEQK IAAIEQK G-NH_2_
CS1-2	Ac-G IAAIEQK IAAIE**W**K **D**AAIEQK IAAIEQK IAAIEQK G-NH_2_
CS1-4	Ac-G IAAIEQK IAAIEQK IAAIEQK IAAIE**W**K **D**AAIEQK G-NH_2_

If these new classes of metallocoiled coils are to reach their full potential as luminescent probes or MRI contrast agents, it is vital to perform a systematic and rigorous study to identify the essential design features for lanthanide coordination, and at the same time, ways to optimize the design in terms of overall stability and MRI relaxivity. Herein, we present a library of new designs, with which we begin to address these issues. For the first time we demonstrate, by systematically moving the binding site linearly along the coiled coil, that the metal coordination chemistry, and in this case the associated luminescent, and more strikingly the MRI properties, are all highly location dependent. In fact translating the binding site 10 Å, enhances MRI relaxivity four-fold compared to our preliminary design, Gd(MB1-2)_3_.^19^

## Results and discussion

### Importance of binding site location along coiled coil

Our original design, MB1-2, features the designed lanthanide binding site between the second and third heptad, however, on closer inspection it is apparent that there are four distinct locations along the coiled coil, at which it could have been introduced. The binding site could be moved up a heptad towards the top (N-terminus of the coiled coil) to yield MB1-1, or down one or two heptads (towards the C-terminus) to yield MB1-3 and MB1-4, respectively (see Fig. 1 and Table 1). At first glance it may appear that the system is symmetrical, leading to two peptides with Asn_3_Asp_3_ binding sites at the extremities of the coiled coil, and two with binding sites located more centrally. However, upon closer inspection, and due to the spatial positioning of the l-amino acid side chains, this is not the case, with for example, the binding site in MB1-1 being located closer towards the coiled coil terminus than in MB1-4, see Fig. 1. Furthermore, in the case of MB1-1 the Asn layer is located more terminally (and more exposed to solvent), whereas in MB1-4 this is instead the Asp layer. So as to establish the importance of binding site location, the complete series was prepared and studied.

### Location dependent coiled coil stability

**Fig. 2:**
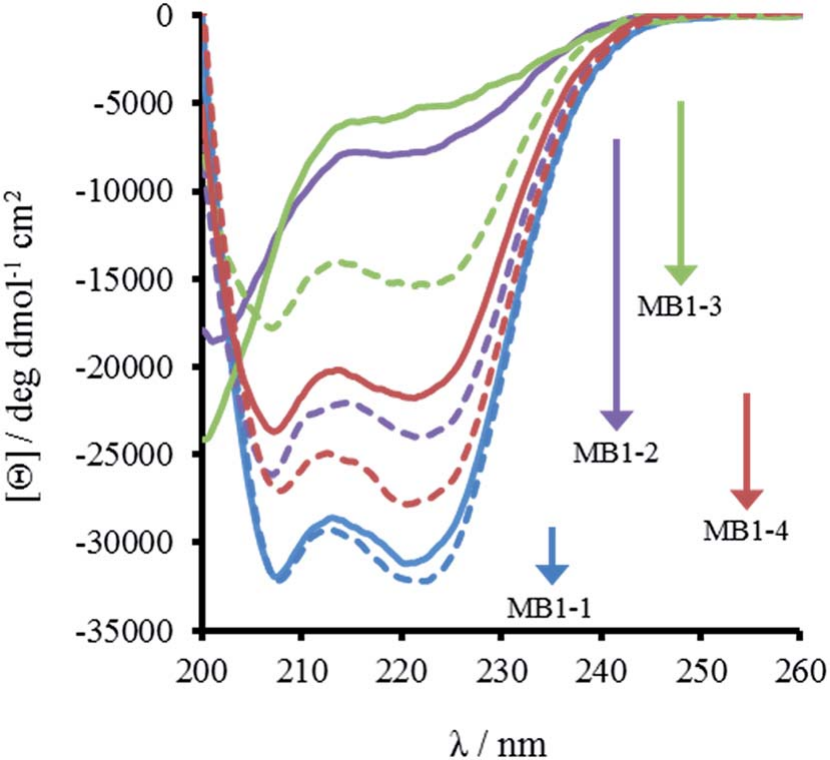
CD spectra of 30 μM MB1-1 (blue), MB1-2 (purple), MB1-3 (green) and MB1-4 (red) peptide monomer, in the absence (solid line) and presence of 10 μM GdCl_3_ (dashed line), recorded at 293 K in 5 mM HEPES buffer pH 7.0.

The most striking difference between these isomeric coiled coils, which consist of the same amino acids located in the same heptad positions, is their difference in folding. A 30 μM solution of MB1-3 monomer in 5 mM HEPES buffer pH 7.0, is similar, though slightly less folded than MB1-2 (15 ± 1 and 21 ± 3%, respectively), see Fig. 2 and Table 2. However, when the binding site is translated closer towards the coiled coil terminus, the apo peptides become increasingly more folded: 55 ± 6% for MB1-4, and 80 ± 6% for MB1-1 which contains the most terminal binding site. These findings suggest that the binding site is more tolerated towards the end of the coiled coil, where the ability to expand and fray may be important in order to adjust and accommodate the potentially bulky site. Furthermore, this observation is consistent with a report that core heptads are three times more stabilizing than N- or C-terminal heptads.^20^

**Table 2:** Summary of % folded values, free energies of folding (
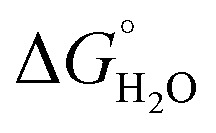
), binding constant (log *K*), number of inner sphere water molecules (#H_2_O) and relaxivity values (*r*_1_ and *r*_2_)

	Apo-% folded^*a*^	Metallo-% folded^*a*^	Apo- 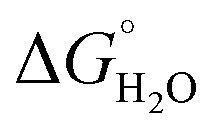 /kcal mol^–1^	Metallo- 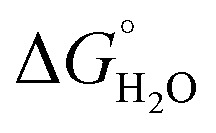 /kcal mol^–1^	log *K*_Tb_	#H_2_O	*r*_1_/mM^–1^ s^–1^	*r*_2_/mM^–1^ s^–1^
MB1-1	80 ± 6	83 ± 7	20.8 ± 3.5	22.4 ± 1.5	5.30 ± 0.15	3.1 ± 0.2	10.0 ± 1.5	89.3 ± 16.8
MB1-2	21 ± 3	62 ± 3	12.7 ± 1.5	15.3 ± 2.0	5.48 ± 0.20	0.0 ± 0.1	4.2 ± 1.2	21.3 ± 2.6
MB1-3	15 ± 1	41 ± 4	—	16.7 ± 3.9	5.16 ± 0.26	0.0 ± 0.1	4.0 ± 1.0	20.9 ± 1.0
MB1-4	55 ± 6	70 ± 5	16.3 ± 2.6	19.3 ± 4.8	5.26 ± 0.36	1.8 ± 0.4	7.5 ± 4.1	37.9 ± 4.0

^*a*^Data reported for 30 μM peptide monomer ± 10 μM GdCl_3_. Analogous data for 5 and 100 μM peptide monomer solutions can be found in the ESI (Table S1).

Importantly, the CD spectra show an increase in folding on addition of up to one equivalent of Gd(iii) per trimer (there is no substantial increase in folding above one equivalent), for all four peptides, see Fig. 2 and S2,† consistent with retention of Gd(iii) binding regardless of binding site location. However, a similar trend was observed with respect to peptide folding on formation of Gd(MB1)_3_: Gd(MB1-3)_3_ is the least well folded (41 ± 4%), followed by Gd(MB1-2)_3_ (62 ± 3%), with Gd(MB1-4)_3_ (70 ± 5%) and Gd(MB1-1)_3_ (83 ± 7%) being the most well folded, see Fig. 2, S2, Tables 2 and S1.†

In order to assess coiled coil stability, rather than simply the extent of folding, both chemical and thermal denaturation studies were performed and monitored by CD. The signal at 222 nm in the CD spectra (an indication of folding) of 30 μM solutions of peptide monomers in 5 mM HEPES buffer pH 7.0, were monitored with increasing urea concentration. Unfolding curves were fit to a two-state equilibrium model between folded trimer and unfolded monomer (see Fig. S3†). The free energies of folding in the absence of denaturant, 
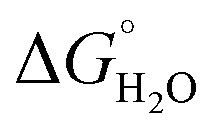
, were determined in the absence and presence of Gd(iii), see Table 2. Though apo-MB1-3 could not be reliably fit due to the lack of a clear baseline for the unfolding transition at low denaturant concentrations, the other values are consistent with MB1-1 being the most stable, followed by MB1-4, MB1-2, with MB1-3 being the least stable. In all four cases, Gd(iii) coordination and Gd(MB1)_3_ assembly was found to be stabilizing. Analogous thermal denaturation studies were monitored by CD, and show a similar trend (see Fig. S4†).

### Peptide oligomerisation

Though these four peptides have been designed to assemble into three stranded coiled coils on coordination of lanthanide ions, this was verified experimentally by performing sedimentation equilibration studies on 100 μM solutions of peptide monomer in the presence of one equivalence of Gd(iii) per trimer, in 10 mM HEPES buffer pH 7.0. A similar approach has been successfully applied in the past to peptide assembling systems.^21^ Equilibrium data yielded weight-average molar masses between 8.6 and 11.2 kDa (see Table S2†). The proportion of trimer was estimated using the expected molar mass of the complex and monomer. MB1-1 and MB1-4 showed a greater proportion of trimer than MB1-2 and MB1-3, consistent with the CD results.

### Lanthanide binding

**Fig. 3:**
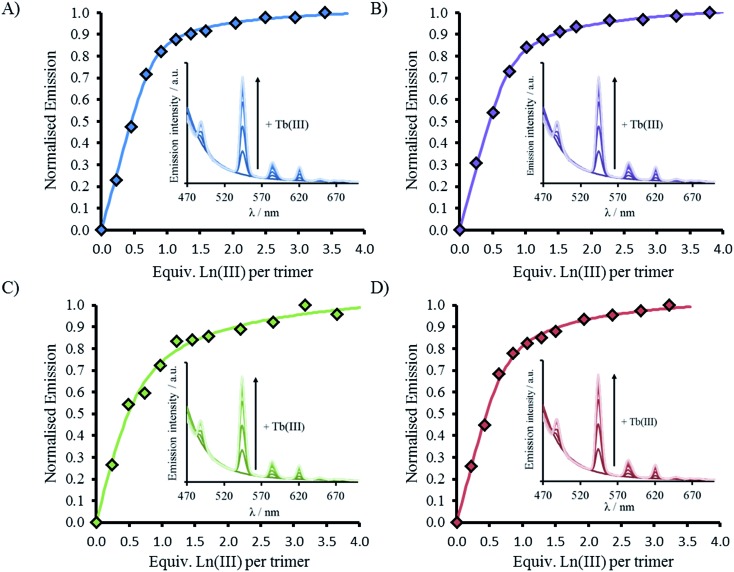
Emission spectra (inset) and plot upon titration of TbCl_3_ into 30 μM peptide monomer for (A) MB1-1 (blue), (B) MB1-2 (purple), (C) MB1-3 (green) and (D) MB1-4 (red). All spectra recorded at 293 K in 10 mM HEPES buffer pH 7.0. Data fit to M + 3L → ML_3_ model using DynaFit. *λ*_exc_ = 280 nm.

CD spectra show an increase in folding on addition of both Gd(iii) and Tb(iii), see Fig. 2, S2 and S5,† but luminescence was subsequently employed to gain insight into the coordination directly using Tb(iii). Addition of up to one equivalence of Tb(iii) per peptide trimer, was accompanied by the appearance of characteristic Tb(iii) emission peaks at 490, 545, 585, 620, and 650 nm (see Fig. 3) following excitation of the Trp unit at 280 nm. This emission is highly sensitized compared to that for Tb(iii) in the absence of peptide. The emission enhancement in all four cases can be attributed to sensitization by the Trp unit on coordination to the designed Asn_3_Asp_3_ binding site, as shown by excitation spectroscopy when the Tb(iii) luminescence signal at 545 nm is monitored (see Fig. S6†).

Plots of the integrated emission intensity over the range 530–560 nm, as a function of Tb(iii) equivalents, are shown in Fig. 3 for all four peptides. These show a sharp increase followed by a plateau at one equivalence of Tb(iii) per three strands of peptide, consistent with formation of the designed Tb(MB1)_3_ species. Data fits to a 1 : 3 Tb(iii):peptide monomer binding model, see Fig. 3. Despite differences in coiled coil folding and stability, the resulting binding constants are extremely similar (or within error), see Table 2.

**Fig. 4:**
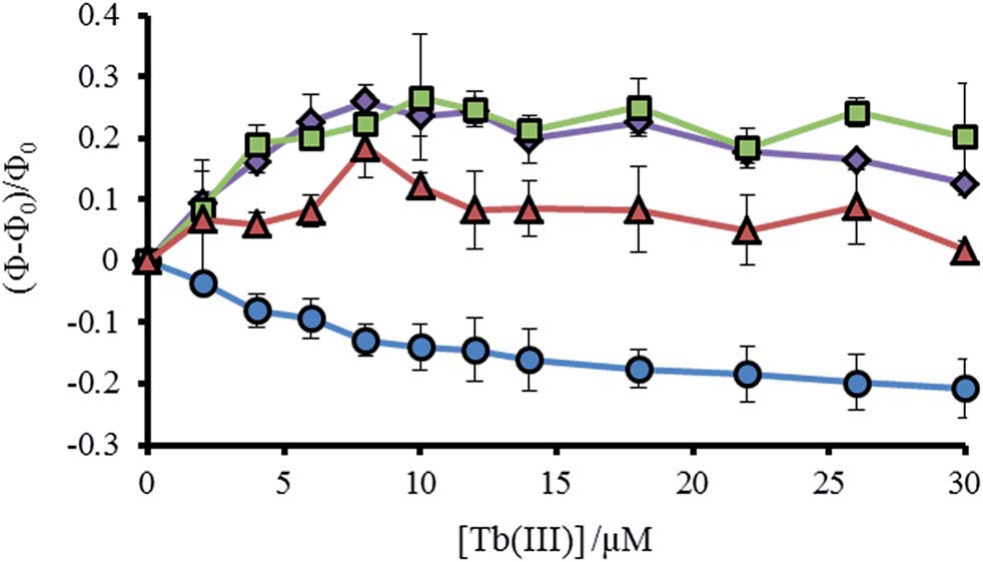
Plot showing the change in tryptophan emission upon titration of TbCl_3_ into 30 μM MB1-1 (blue circles), MB1-2 (purple diamonds), MB1-3 (green squares) and MB1-4 (red triangles) peptide monomer, at 293 K in 10 mM HEPES buffer pH 7.0. Change in emission calculated from integration of the emission peak (*Φ*) between 305–450 nm, where *Φ*_0_ is the integration in the absence of Tb(iii). *λ*_exc_ = 280 nm.

As noted previously, a decrease is not observed, but rather an increase, in the Trp emission signal (305–450 nm) on addition of Tb(iii) to a solution of MB1-2, which we attributed to a structural change on folding and an associated change in Trp environment.^19^ This behavior is mirrored by the similar MB1-3 peptide. However, for MB1-4, which shows substantially improved folding in the absence of Tb(iii) and therefore a more modest change on Tb(iii) binding, the Trp emission signal shows only a modest change. In complete contrast, our most folded assembly, which shows very little change on Tb(iii) binding, MB1-1, does not show an increase in Trp emission, but rather shows a decrease. Due to the lack of a substantial structural change (see Fig. S5†), this decrease could be assigned to energy transfer on sensitizing the Tb(iii) emission (see Fig. 4 and S7†).

**Fig. 5:**
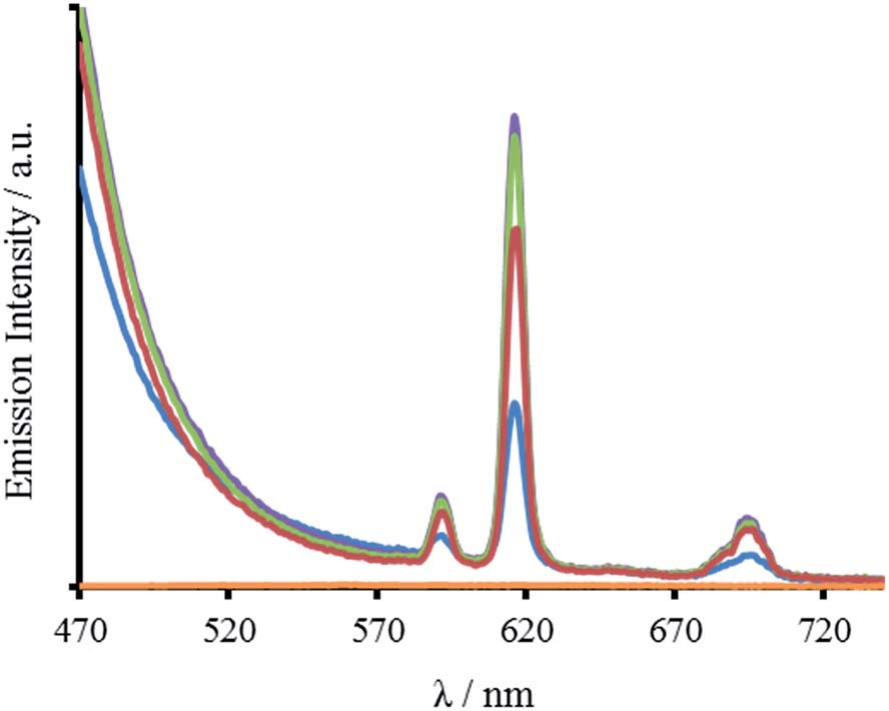
Emission spectra of 30 μM MB1-1 (blue), MB1-2 (purple), MB1-3 (green), MB1-4 (red) monomer peptide, and a blank (orange), in the presence of 10 μM EuCl_3_, recorded at 293 K in 10 mM HEPES buffer pH 7.0. *λ*_exc_ = 280 nm.

In addition to Tb(iii), the Trp can also sensitize Eu(iii) emission. Spectra recorded for all four peptides in the presence of Eu(iii) display characteristic Eu(iii) emission profiles (Fig. 5 and S8). As for the Tb(iii) spectra, the emission enhancement is attributed to Eu(iii) binding in close proximity to the Trp, and to the designed site. The lack of any observable ^5^D_0_ → ^7^F_0_ transition at 580 nm and the increased intensity of the *j* = 2 over the *j* = 1 emission (8.8 times larger) for all four peptides, indicate a symmetric Eu(iii) site, consistent with binding to a three-fold symmetric coiled coil.^22^–^24^

### Location dependent lanthanide hydration

Though all four of our designs, MB1-1, MB1-2, MB1-3 and MB1-4, bind lanthanide ions, as is evident from induced structural changes on lanthanide binding, as well as sensitized Tb(iii) and Eu(iii) luminescence, both the apo and metallo-peptides display different stabilities, ascribed to the different location of the binding site along the coiled coil. We, therefore, set out to interrogate the lanthanide coordination chemistry more thoroughly at these four different sites. Specifically we wished to determine if exogenous water molecules were able to coordinate to the bound Tb(iii) in any of the four Tb(MB1)_3_ structures. Differences could arise due to altered peptide stabilities and therefore increased water penetration, though more likely are that differences in local peptide structure (fraying and unwinding/folding) and ease of water penetration at either termini of the coiled coil (MB1-1 and MB1-4), will have a bigger impact on the Tb(iii) hydration state.

Luminescence lifetime decay studies of the Tb(MB1)_3_ peptides in H_2_O and D_2_O, monitored at 545 nm, were performed, and rates of decay were input into the Parker–Beeby equation,^25^ in order to estimate the amount of inner sphere water bound to the coordinated Tb(iii). Despite being less folded and stable, the Tb(MB1-3)_3_ complex was found to have no (0.0 ± 0.1) inner sphere water coordinated to the bound Tb(iii), see Table 2, which is the same as we previously reported for the similar MB1-2 analogue.^19^ This observation is consistent with both of these peptides providing all nine donor atoms, as well as binding to a site generated centrally within the hydrophobic core of a coiled coil. However, inner sphere water was found to be present when Tb(iii) coordinates more towards the terminus of a coiled coil, as is the case for both MB1-1 and MB1-4. The Tb(MB1-1)_3_ and Tb(MB1-4)_3_ complexes were found to have three (3.1 ± 0.2) and two (1.8 ± 0.4) inner sphere water molecules, respectively, see Table 2.

### Evaluating essential residues for lanthanide binding

Here we report a thorough and complete study which examines all of the possible binding sites within our coiled coil, is the only example which translates complex binding sites, with multiple residues contributing to the first coordination sphere, and is the only example in which the integer number of water molecules coordinated is dependent on binding site location. These findings are in line with a previous report which found greater water access when a Zn(His)_3_ site was moved from the C- to the N-terminus of a coiled coil, and decreased water access when located more within the middle;^14^ as well as a report from which small fractional differences in the extent of inner sphere water can be inferred,^26^ and related physical properties,^27^ for Cd sites along a coiled coil. However, in all of these examples, regardless of metal site location, the same amino acid side chains remained coordinated to the metal ion. In contrast, our findings suggest that either some or all of the Asp residues could be coordinated in a monodentate, rather than a bidentate fashion, or that not all Asn and Asp residues are always essential for binding. We hypothesized that the latter may be the case in Tb(MB1-1)_3_, where the Asp layer is coordinated to the Tb(iii) in a bidentate fashion, but where the Asn residues are not engaged in Tb(iii) coordination. Instead the Asn residues may form a hydrogen bonded triangular network, aided by coiled coil fraying and expansion at the N-terminus, thereby permanently vacating three coordination sites which can then be populated by three inner sphere water molecules, see Fig. 1. We therefore set out to fully interrogate the coordination requirements of the site for lanthanide binding, and prepared a series of related peptides which lack the top Asn layer, but retained the lower Asp layer, to yield CS1-1 (a N-terminal site), CS1-2 (a central site) and CS1-4 (a C-terminal site), respectively (see Table 1).

**Fig. 6:**
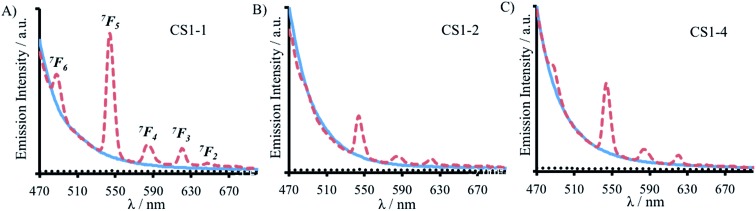
Emission spectra of 30 μM (A) CS1-1, (B) CS1-2 and (C) CS1-4 monomer peptide, in the absence (solid blue line) and presence of 10 μM TbCl_3_ (dashed red line), recorded at 293 K in 10 mM HEPES buffer pH 7.0. *λ*_exc_ = 280 nm.

A 30 μM solution of CS1-2 monomer is exceedingly better folded in the absence of a lanthanide than the related MB1-2 peptide (51% compared to 21 ± 3%), see Fig. S9B and Table S1,† consistent with the introduction of fewer destabilizing polar residues within the hydrophobic core. The addition of one equivalence of Tb(iii) per trimer, was accompanied by only a small increase in folding (from 51 to 59%, see Fig. S9B†), whereas MB1-2 shows a greater change in folding (from 20 to 57%) on formation of Gd(MB1-2)_3_. An analogous luminescence study showed, that whereas on formation of Tb(MB1-2)_3_ we observed notably sensitized Tb(iii) luminescence (see Fig. 3B),^19^ only a modest increase is observed in the presence of CS1-2 (see Fig. 6B). The latter could be consistent with non-specific Tb(iii) binding, to a combination of the Asp (located adjacent to the Trp) and Glu residues. Similarly, Tb(iii) binding has less impact on the luminescence (Fig. 6C) and CD (Fig. S9C†) spectra of CS1-4 compared to MB1-4. These observations would be consistent with both the Asn and the Asp residues being important for lanthanide binding in these two sites. Therefore, in the case of Tb(MB1-4)_3_, the two water molecules are likely to coordinate to vacant sites due to Tb(iii) coordination by only some of the Asn and Asp O-donors, see Fig. 1.

In stark contrast to both CS1-2 and CS1-4, Tb(iii) binding to CS1-1 resulted in strongly sensitized luminescence (see Fig. 6A), consistent with retention of binding despite the lack of an Asn layer. Data from this titration could be fit to a 1 : 3 Tb(iii) : CS1-1 monomer binding model, see Fig. S10,† to yield a log *K*_Tb_ of 4.57 ± 0.07. Luminescence decay studies of the resulting Tb(CS1-1)_3_ complex were consistent with a similar inner sphere water content (3.6 ± 0.1) compared to the MB1-1 analogue, and sedimentation equilibrium studies confirmed the formation of a trimer (see Table S2†). These findings are in agreement with our hypothesis that the Asn residues are not essential, and are not involved in Tb(iii) coordination, in Tb(MB1-1)_3_, see Fig. 1. These Ln(iii) sites are likely to be dynamic and Asn residues may to a small extent occasionally contribute to the Ln(iii) coordination sphere, which may account for the, though similar, slightly lower binding constant for CS1-1 compared to MB1-1. Therefore, the similar binding constants obtained for Tb(iii) binding to MB1-1 and MB1-2 (see Table 2) despite fewer proposed peptide donor ligands, we suggest to be due to enhanced peptide self association affinity, which has previously been reported to enhance metal binding affinity,^28^ compensating for the formation of what would be expected to be a less stable metal coordination environment, Tb(Asp)_3_(H_2_O)_3_. The relationship between metal binding site affinity and peptide/protein multimer stability is a theme more widely adopted in metallo-peptide/protein design.^29^

Importantly, CS1-1 has demonstrated that it is possible to coordinate Tb(iii) within a three stranded coiled coil using only an Asp layer, which provides no more than six potential donor oxygens. However, this is highly sensitive to the location of this layer (even within a parallel homotrimer), and only appears to be capable of Tb(iii) coordination when located towards the N-terminus of the coiled coil. To the best of our knowledge, this represents the first report of coordination chemistry requirements being dependent on the metal-site location (of otherwise identical sites) along a coiled coil.

### Location dependent impact on MRI

We previously reported, that despite Gd(MB1-2)_3_, having no inner sphere water molecules, that the relaxivity was still greater than that for the small molecule, clinically adopted, Dotarem® (GdDOTA), under the same experimental condition (300 MHz, 7 T).^19^ This, we hypothesized, to be due to a primarily outer sphere mechanism, which likely involves a hydrogen bonding network between outer sphere water molecules and the coiled coil surface, reduced tumbling in solution due to the coiled coils greater size, and proton exchange between peptide and bulk water. Considering the different chemistries, specifically with respect to the extent of inner sphere water, when the location of the Gd(iii) binding site is translated along the coiled coil, we set out to evaluate their efficiency as MRI contrast agents. Therefore, MR images (*T*_1_ and *T*_2_ maps) were recorded of phantom samples containing increasing concentrations of Gd(MB1)_3_ in 10 mM HEPES buffer pH 7.0, see Fig. S11.† The gradient of a plot of the inverse of the *T*_1_ and *T*_2_ water relaxation times, as a function of Gd(MB1)_3_ concentration, yields the longitudinal (*r*_1_) and transverse (*r*_2_) relaxivity (mM^–1^ s^–1^), a measure of the efficiency of the complex to alter the relaxation time of bulk water, see Fig. S12.†

The Gd(MB1-3)_3_ complex displayed comparable longitudinal (*r*_1_ = 4.0 ± 1.0 mM^–1^ s^–1^) and transverse (*r*_2_ = 20.9 ± 1.0 mM^–1^ s^–1^) relaxivity compared to the MB1-2 analogue (*r*_1_ = 4.2 ± 1.2 mM^–1^ s^–1^; *r*_2_ = 21.3 ± 2.6 mM^–1^ s^–1^), see Table 2. These observations are consistent with similar coordination chemistries and structures. In contrast, both the longitudinal (*r*_1_ = 7.5 ± 4.1 mM^–1^ s^–1^) and transverse (*r*_2_ = 37.9 ± 4.0 mM^–1^ s^–1^) relaxivity of Gd(MB1-4)_3_ are notably larger, consistent with a contribution from both an outer and inner sphere mechanism. Not surprisingly, increasing the inner sphere water content from two (MB1-4) to three water molecules (MB1-1) leads to the Gd(MB1-1)_3_ complex being the most efficient at altering the relaxation time of bulk water (*r*_1_ = 10.0 ± 1.5 mM^–1^ s^–1^; *r*_2_ = 89.3 ± 16.8 mM^–1^ s^–1^), see Table 2.

These findings demonstrate, that the location of the Gd(iii) binding site within the coiled coil can critically alter the relaxivity of the agent, by bringing into play multiple mechanisms by which magnetization is transferred to bulk water protons. As a result, we now have a library of complexes with longitudinal relaxivity ranging from 4–10 mM^–1^ s^–1^, and transverse relaxivity, on which these Gd(MB1)_3_ complexes appear to have a more pronounced effect at 7 T, from 21–89 mM^–1^ s^–1^.

These large increases in relaxivity, achieved by translating the binding site a single heptad, is unprecedented, and complexes such as these may therefore warrant further investigation as possible MRI contrast agents, if the limitations with respect their stability can be overcome. The goal of this work has been to interrogate a new class of lanthanide complexes, and to identify potential areas in which they could be applied, such as MRI, but this report is not advocating that the gadolinium complexes presented herein should find their way into a clinical setting.

## Conclusions

This study illustrates the level of control that can now be achieved in *de novo* metal binding site design, by, for the first time demonstrating that translation of a designed lanthanide binding site (with two layers of coordinating residues) along a coiled coil scaffold, yields peptide architectures with radically different degrees of folding and stabilities; and more importantly, that the resulting metal coordination chemistry, and ligand requirements, is highly dependent on the binding site location. Harnessing this knowledge is essential for the successful design of functional metallo peptides, where desirable properties can require a fine balance between protein folding, stability and metal coordination chemistry. In this report, we have focused on the coordination of non-biological *xeno* metal ions to *de novo* peptide scaffolds, for non-biological applications. By careful selection of binding site location, and using the herein established design rules, we have designed lanthanide coiled coils that may be more suitable for luminescence studies (MB1-2 and MB1-3, no inner sphere water), or for MR imaging applications (MB1-1, three inner sphere water molecules). Notably, translating the binding site a single heptad (*ca.* 10 Å) towards the N-terminus, switches the design between these two extremes, and leads to a four-fold increase in MRI relaxivity. These designs which incorporate *xeno* metals, may be of interest as potential novel imaging agents, but more importantly, they provide a greater understanding of the importance of binding site location and how that impacts on both protein stability and coordination chemistry. We are therefore now one step closer to the truly *de novo* design of coordination sites with predictable properties and chemistries, for these, and currently unforeseen applications.

## Materials and methods

The following reagents were purchased from Sigma Aldrich: GdCl_3_·6H_2_O, EuCl_3_·6H_2_O and TbCl_3_·6H_2_O. Urea (≥99% purity), xylenol orange indicator, glacial acetic acid and 4-(2-hydroxyethyl)-1-piperazineethanesulfonic acid (HEPES) were all purchased from Fisher Scientific Ltd. All Fmoc protected amino acids, dimethylformamide (DMF) and *N*,*N*,*N*′,*N*′-tetramethyl-*O*-(1*H*-benzotriazol-1-yl)uronium hexafluorophosphate (HBTU) were purchased from Pepceuticals Ltd, Leicester. The rink amide MBHA resin was obtained from AGTC Bioproducts Ltd, along with diisopropylethylamine (DIPEA) and trifluoroacetic acid (TFA). All D_2_O was purchased from VWR and the Gd(iii) and Tb(iii) standards from SCP Science, Quebec.

### Peptide synthesis and purification

Peptides were synthesized on a CEM Liberty Blue automated peptide synthesizer on rink amide MBHA resin (0.25 mmol scale, 0.65 mmol g^–1^), using standard Fmoc-amino acid solid-phase peptide synthesis protocols^30^ and purified and characterized as previously reported.^31^

### Sample preparation

Stock solutions of GdCl_3_, TbCl_3_ and EuCl_3_ (1 mM) were prepared in deionized water, and their accurate concentrations determined spectroscopically using xylenol orange indicator and Ln(iii) standard solutions as reported by Fedeli *et al.*^32^ Peptide concentrations were determined based on the tryptophan absorbance at 280 nm (*ε*_280_ = 5690 M^–1^ cm^–1^) in 7 M aqueous solutions of urea.

### Circular dichroism (CD) spectroscopy

CD spectra for solutions containing 30 and 100 μM monomer in 5 mM HEPES buffer pH 7.0, were recorded in a 1 mm path length quartz cuvettes on a Jasco J-715 spectropolarimeter. The observed ellipticity in millidegrees was converted into molar ellipticity, (*Θ*), and is reported in units of deg dmol^–1^ cm^2^. The percentage folding, %_folded_, was calculated based on the theoretical maximum ellipticity value of –39 054 deg dmol^–1^ cm^2^ at 222 nm (eqn (1)), based on reports by Scholtz *et al.*^33^1
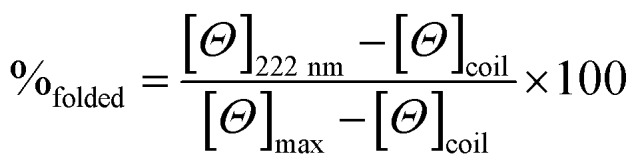



The maximum ellipticity, [*Θ*]_max_, is determined from (–42, 500 × (1 – (3/*n*))), where *n* is the number of residues in the sequence, and [*Θ*]_coil_ is the ellipticity of a random coil.^19^ Solutions of metallo coiled coils were prepared on addition of aliquots of 1 mM stock solutions of GdCl_3_ or TbCl_3_. The percentage folded values for the apo and metallo peptides were calculated from an average of three repeats, and the standard deviation reported.

Chemical unfolding data was recorded by monitoring the ellipticity at 222 nm of a 30 μM solution of peptide monomer in 5 mM HEPES buffer pH 7.0 in the absence and presence of 10 μM GdCl_3_, as a function of urea concentration (from 0 → 6.5 M). The chemical denaturation data was fit to a two-state, folded to three monomers, equilibrium model using global analysis nonlinear least squares fitting in MATLAB as outlined in the procedure by Buer *et al.*^34^ Thermal unfolding experiments were recorded using a Jasco Peltier temperature accessory, over the temperature range 20–90 °C, with a temperature gradient of 0.38 °C min^–1^, whilst monitoring the signal at 222 nm.

### Luminescence

Emission spectra were recorded in a 1 cm path length quartz cuvette using an Edinburgh Instruments Fluorescence FLS920 system with a 450 W xenon arc lamp and a Hamamatsu R928 photomultiplier tube. The emission monochromator was fitted with two interchangeable gratings blazed at 500 nm and 1200 nm and the data was collected using F900 spectrometer analysis software. Aliquots of a 1 mM stock solution of TbCl_3_ or EuCl_3_ were titrated into 30 μM peptide monomer solutions in 10 mM HEPES buffer pH 7.0, and the emission profile recorded after 20 min equilibration. Solutions were excited at 280 nm and the emission was scanned from 305–450 nm (Trp region) or 475–750 nm (Ln(iii) region) using 305 and 455 nm long pass filters, respectively. Spectra were corrected for instrument response (grating/PMT) in all cases. The integration of the tryptophan emission was measured between 305–350 nm; the Tb(iii) emission integration at 545 nm was measured between 530–560 nm; and the Eu(iii) emission integration at 591 and 616 nm were measured between 581–598 nm, and 605–629 nm, respectively. The Tb(iii) emission integration data was fit using DynaFit Software (Biokin Ltd, Massachusetts)^35^ with a M + 3L → ML_3_ (*K*_1_) binding equation to determine the binding constant. This was converted to a first order binding constant based on ⅓M + L → ⅓(ML_3_) (*K*), where 
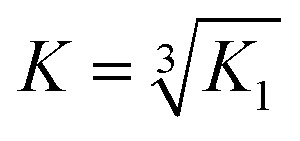
. The model included a variable monomer concentration to account for any errors in sample preparation, and led to values of 27.5, 25.0, 29.0 and 27.8 μM for MB1-1, MB1-2, MB1-3 and MB1-4, respectively.

Tb(iii) lifetimes in D_2_O and H_2_O were determined for all Tb-peptide complexes by monitoring solutions containing 10 μM Tb(iii) and 100 μM monomer peptide (to ensure >99% of the Tb(iii) is bound) in 10 mM HEPES buffer pH 7.0 using a μF flash lamp light source (50 Hz) on an Edinburgh Instruments spectrofluorimeter, collecting over a 10 ms time range, with a lamp trigger delay of 0.1 ms. The peptide samples were deuterated by equilibration in 99.9% D_2_O for 8 hours prior to lyophilisation. This process was repeated and then the lifetime of the deuterated samples recorded in 99.96% D_2_O. Data was fitted to mono-exponential decay kinetics in Kaleidagraph using the Marquardt–Levenberg linear least squares algorithm, and from the observed lifetime the number of coordinated water molecules was determined using the Parker–Beeby equation.^25^ The absorption spectra of the excitation samples were recorded on a Shimadzu AP-120 photometer between 200 and 400 nm.

### NMR spectroscopy

Data was collected on a Bruker DMX 300 spectrometer equipped with a 7 T vertical wide-bore superconducting magnet operating at a proton resonance frequency of 300.13 MHz with a 30 mm RF bird cage coil. The spectrometer was controlled by a Linux workstation operating TOPSPIN version 1.3 and ParaVision version 3.0 software. All experiments were recorded at 293 K and the 90° and 180° radiofrequency pulses were calibrated for each sample. *T*_1_ and *T*_2_ maps of water protons in phantom samples prepared in 5 mm NMR tubes were acquired using a RARE (Rapid Acquisition with Relaxation Enhancement) spin-echo imaging sequence. Horizontal images were acquired with a 1 mm slice thickness, using a 25 × 25 mm field-of-view and a 64 × 64 pixel matrix. A repetition time of *T*_R_ = 16 s was used. *T*_1_ relaxation maps were produced from a series of 9 spin echo images with varying *T*_1_ inversion recovery delays from 2.2–15 000 ms and RARE factor of 8. *T*_2_ relaxation maps were produced from 128 echo images with echo times from 10–1280 ms and a RARE factor of 1. All imaging experiments were performed in triplicate and were analyzed using Prospa software (Magritek, Wellington, New Zealand), where the relaxation time for each concentration was taken from the average value from the pixels within the sample. The values for *T*_2_ were corrected for effects of signal loss from diffusion during the imaging sequences (see ESI for details†). The relaxivity (mM^–1^ s^–1^) was calculated from the gradient of a plot of the average 1/*T*_(1,2)_ against the GdCl_3_ concentration. The relaxivity of Gd(MB1-1)_3_, Gd(MB1-2)_3_, Gd(MB1-3)_3_ and Gd(MB1-4)_3_ was determined from solutions containing 20, 30 and 40 μM GdCl_3_ and 6 equivalences of peptide monomer, prepared in 10 mM HEPES pH 7.0. Additional samples of 10 mM pH 7.0 HEPES and 0.1 mM GdCl_3_ were included as controls.

The excess of peptide (6 monomers per Gd(iii)) was used to ensure that >99% of Gd(iii) was bound at the lowest concentration (20 μM). Based on the lowest binding constant determined (*vide supra*) (log *K* = 5.16), we would predict that 99% of the Gd(iii) is bound in the presence of two equivalences of trimer, and that this increases to 99.9% upon addition of the third equivalence of trimer. Addition of a third equivalence was found to have no notable change at 10 μM Gd(iii) (see Fig. S11 and Table S3†).

### Sedimentation equilibrium

Samples were analysed using a Beckman Optima XL-I Analytical Ultracentrifuge at 45 000 rpm (150 000 × *g*). Equal volumes of buffer and peptide solution (100 μM monomer and 1/3 equivalence of GdCl_3_) at 0.7 AU were loaded into cells constructed with 12 mm path length, aluminum epoxy centerpieces and sapphire windows. Absorbance optics at 280 nm were employed and scans performed every hour to observe the approach to equilibrium. Scans were acquired and logged using Proteome Software v6 (Beckman, Palo Alto, CA). Once all samples were confirmed to have reached equilibrium, five scans were obtained in succession.

These final five scans were averaged and analyzed using SEDFIT-MSTAR.^36^ Apparent, weight-average molar masses were obtained through the M* function of Creeth and Harding,^37^ combined with the c(M) method of Schuck *et al.*^35^ to find the meniscus concentration and baseline. These values were assumed to be free from non-ideality due to the low concentration and low monomer molar mass.^36^

## Supplementary Material

Supplementary information
