# Supplementary material for: Location dependent coordination chemistry and MRI relaxivity, in de novo designed lanthanide coiled coils
Source: Chem Sci. 2015 Dec 22;7(3):2207–16. doi: 10.1039/c5sc04101e (PMC5968752; doi:10.1039/c5sc04101e)
Supplement: Supplementary file 1 [file SC-007-C5SC04101E-s001.pdf]

Electronic Supplementary Material (ESI) for Chemical Science.
This journal is © The Royal Society of Chemistry 2016

Supporting Information

Location dependent coordination chemistry and MRI relaxivity, in *de novo* designed lanthanide coiled coils

Matthew R. Berwick[†], Louise N. Slope[†], Caitlin F. Smith[†], Siobhan M. King[†], Sarah L. Newton[†], Richard B. Gillis[‡¥], Gary G. Adams[‡¥], Arthur J. Rowe[‡], Stephen E. Harding[‡], Melanie M. Britton[†], Anna F. A. Peacock[†]*

[†]*School of Chemistry, University of Birmingham, Edgbaston, B15 2TT, UK*

[‡]*National Centre for Macromolecular Hydrodynamics, School of Biosciences, University of Nottingham, Sutton Bonington, LE12 5RD, UK*

[¥] *School of Health Sciences, The University of Nottingham, Queen's Medical Centre, Nottingham, NG7 2HA, UK*

*To whom correspondence should be addressed. E-mail: a.f.a.peacock@bham.ac.uk

Contents:

1. Methods

   Figure S1 – RARE imaging pulse sequence
2. Table S1 – Table of percentage folded values
3. Table S2 – Sedimentation equilibrium results
4. Table S3 – Relaxivity values upon addition of extra trimer equivalence
5. Figure S2 – CD Gd(III) titration
6. Figure S3 – CD chemical denaturation
7. Figure S4 – CD thermal unfolding
8. Figure S5 – CD Tb(III) titration
9. Figure S6 – Excitation and absorption profiles
10. Figure S7 – Tryptophan emission spectra
11. Figure S8 - Luminescence titration of CS1-1
12. Figure S9 - CD spectra of CS1-1, CS1-2 and CS1-4

13. Figure S10 – Emission spectra in H_2O and D_2O

14. Figure S11– Magnetic resonance imaging of phantom samples

15. Figure S12 – Relaxivity plots

16. Figure S13 – HPLC and mass spectrum for MB1-1

17. Figure S14 – HPLC and mass spectrum for MB1-2

18. Figure S15 – HPLC and mass spectrum for MB1-3

19. Figure S16 – HPLC and mass spectrum for MB1-4

20. Figure S17 – HPLC and mass spectrum for CS1-1

21. Figure S18 – HPLC and mass spectrum for CS1-2

22. Figure S19 – HPLC and mass spectrum for CS1-4

23. References

1. Methods

Chemical Denaturation. Chemical unfolding data was recorded by monitoring the ellipticity at 222 nm of a 30 µM solution of peptide monomer in 5 mM HEPES buffer pH 7.0 in the absence and presence of 10 µM $GdCl_3$, as a function of urea concentration (from 0 → 6.5 M).

$$Folded\ (F) \leftrightarrow 3\ Unfolded\ (U) \qquad Eq. 1$$

The chemical denaturation data was fit to a two-state folded to three monomers equilibrium model (see equation 1) as outlined by B. Buer.[1]

Diffusion Corrected T_2. When measuring the T_2 of a sample using a RARE imaging sequence (see Figure S1) the application of a gradient (g_R) in the read direction with duration (2α) leads to signal attenuation due to the effects of water self-diffusion.[2] Therefore, the T_2 observed (T_{2obs}) has a diffusion weighted component (T_{2d}). The correct T_2 value (T_{2corr}) can be determined using Equations 2 and 3, where γ is the proton gyromagnetic ratio, T_E is the experiment echo time and D is the diffusion coefficient of the water. The value of D was approximated to be the diffusion of water at 293 K.[3] An analogous expression exists for the effect of the slice gradient on the relaxation time and was employed in the correction as well.

$$\frac{1}{T_{2obs}} = \frac{1}{T_{2corr}} + \frac{1}{T_{2d}} \qquad Eq. 2$$

$$\frac{1}{T_{2d}} = \gamma^2 g_R^{\ 2} \alpha^2 D \left(\frac{T_E - \frac{4}{3}\alpha}{T_E} \right) \qquad Eq. 3$$

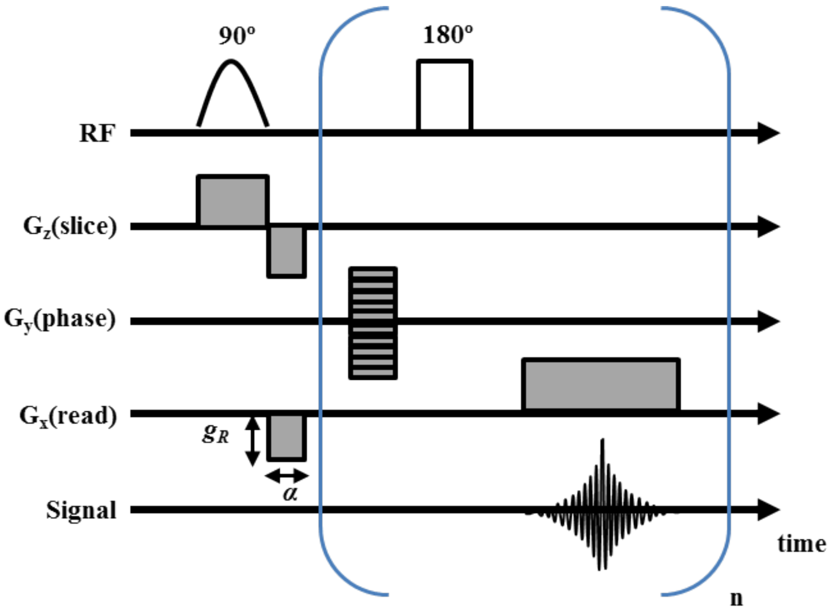

Figure S1. RARE imaging pulse sequence.

2. Table S1: Percentage folded as a function of peptide monomer concentration

	Monomer Conc / µM	Apo % Folded	Metallo % Folded
**MB1-1**	5	65 ± 4	66 ± 6
	30	80 ± 6	83 ± 7
	100	85 ± 3	90 ± 3
**MB1-2**	5	14 ± 2	22 ± 5
	30	21 ± 3	62 ± 3
	100	33 ± 9	69 ± 7
**MB1-3**	5	11 ± 1	13 ± 2
	30	15 ± 1	41 ± 4
	100	16 ± 2	56 ± 5
**MB1-4**	5	43 ± 2	48 ± 3
	30	55 ± 6	70 ± 5
	100	60 ± 6	76 ± 7

All samples recorded in 5 mM HEPES buffer pH 7.0 at 293 K, in the absence or presence of 1/3 equivalence of $GdCl_3$, and molar ellipticity at 222 nm converted to percentage folded. Average value taken from three repeats and reported with standard deviations.

3. Table S2. Weighted average molar mass, and calculated proportion of trimer, based on sedimentation equilibrium experiments performed in the presence of 1 equiv. Gd(III) per three strands of peptide monomer.

Peptide	Weight-average molar mass / kDa	Proportion of trimer complex / %*
MB1-1	10.6 ± 0.6	81 ± 7
MB1-2	9.0 ± 0.5	61 ± 6
MB1-3	8.6 ± 0.4	56 ± 5
MB1-4	10.9 ± 0.6	84 ± 8
CS1-1	11.2 ± 0.6	88 ± 7

*Assuming only presence of monomer and trimer

4. Table S3. Average 1/*T*1 and 1/*T*2 for 0.01 mM Gd(III) in the presence of two equivalence of peptide trimer, and on addition of a third equivalence.

**Peptide**	**$(1/T_1)$ / s^{-1}**	**$(1/T_1)$ third equivalence / s^{-1}**	**$(1/T_2)$ / s^{-1}**	**$(1/T_2)$ third equivalence / s^{-1}**
MB1-1	0.44 ± 0.03	0.45 ± 0.02	1.31 ± 0.03	1.35 ± 0.14
MB1-2	0.41 ± 0.01	0.41 ± 0.01	0.95 ± 0.02	0.93 ± 0.03
MB1-3	0.40 ±0.01	0.40 ± 0.01	0.93 ± 0.05	0.96 ± 0.03
MB1-4	0.43 ± 0.02	0.40 ± 0.02	1.01 ± 0.08	0.98 ± 0.10

5. Figure S2:

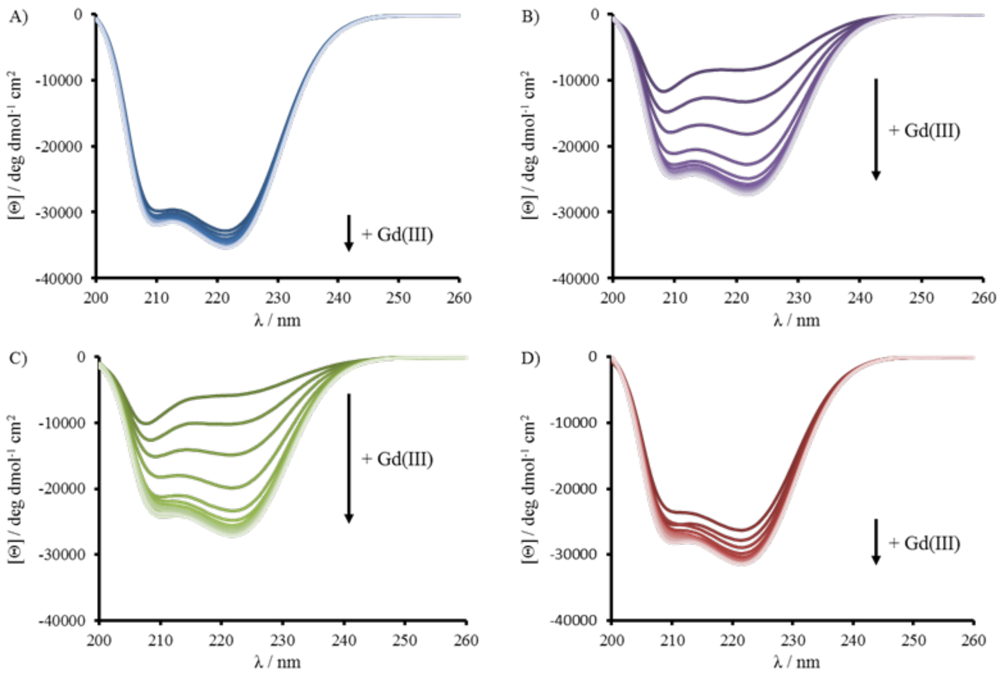

Figure S2. $GdCl_3$ titration into 100 μM A) MB1-1, B) MB1-2, C) MB1-3 and D) MB1-4 peptide monomer in 5 mM HEPES buffer pH 7.0, monitored by CD, going from 0 μM to 100 μM Gd(III).

6. Figure S3:

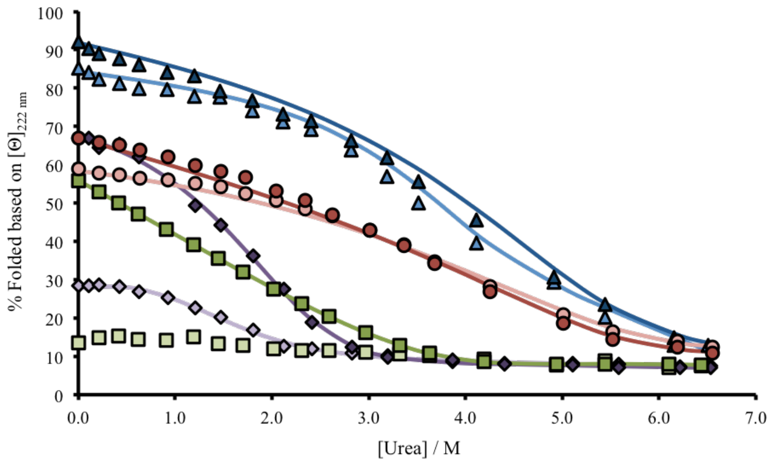

Figure S3. Urea chemical denaturation of 30 μM monomer MB1-1 (blue triangles), MB1-2 (purple diamonds), MB1-3 (green squares) and MB1-4 (red circles) in the absence (light color) and presence (dark color) of 10 μM Gd(III). Recorded in 5 mM HEPES buffer pH 7.0, monitored by CD at 222 nm, and fit to a trimer ↔ three monomer equilibrium using a nonlinear least-squares fitting (no fit was obtained for apo MB1-3).

7. Figure S4:

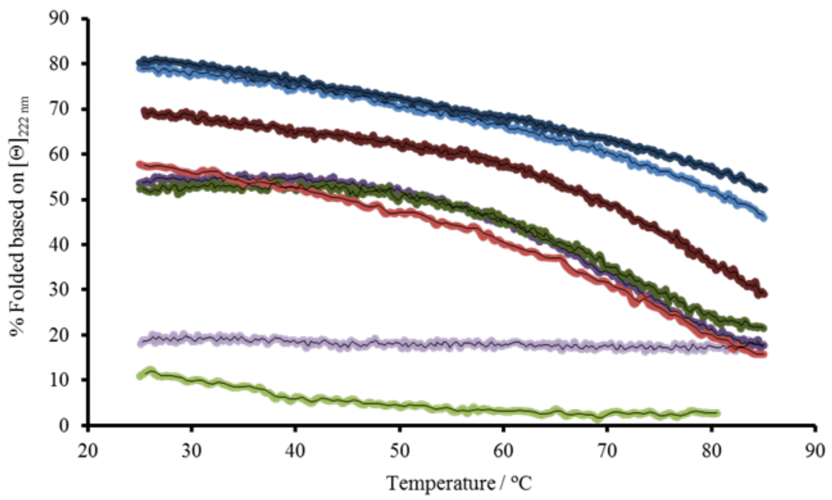

Figure S4. CD thermal unfolding of 30 µM MB1-1 (blue), MB1-2 (purple), MB1-3 (green) and MB1-4 (red) peptide monomer, in the absence (light color) and presence (dark color) of 10 µM $GdCl_3$. Recorded in 5 mM HEPES buffer pH 7.0.

8. Figure S5:

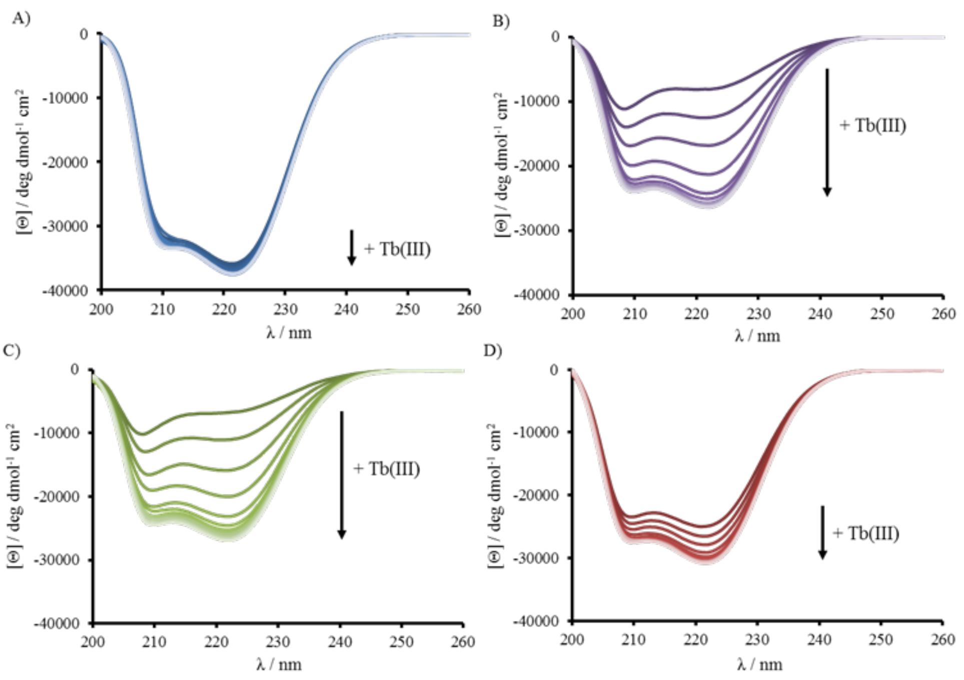

Figure S5. $TbCl_3$ titration into 100 µM A) MB1-1, B) MB1-2, C) MB1-3 and D) MB1-4 peptide monomer in 5 mM HEPES buffer pH 7.0 monitored by CD, going from 0 µM to 100 µM Tb(III).

9. Figure S6:

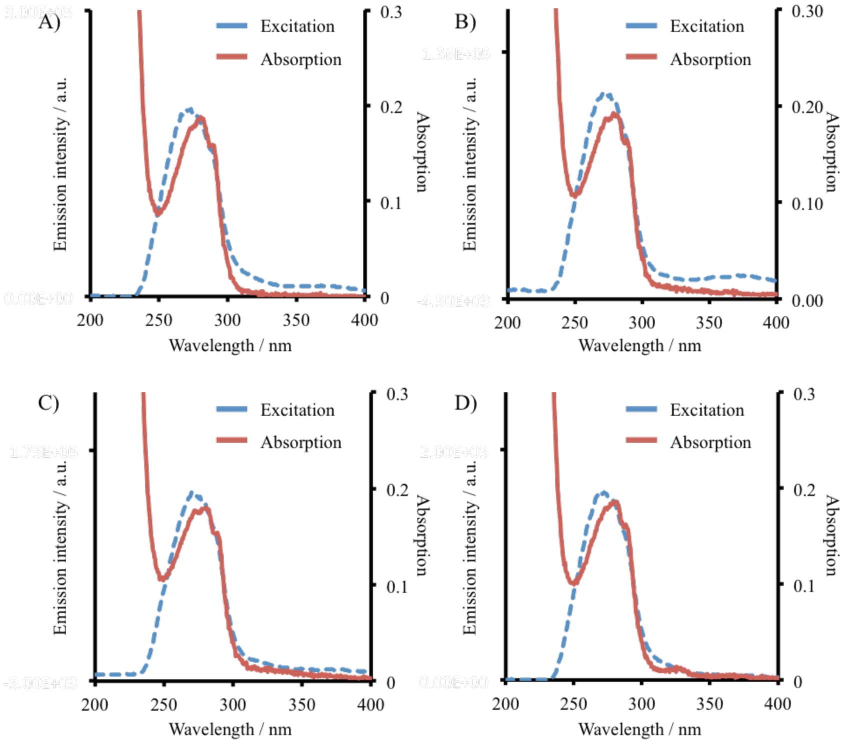

Figure S6. Absorption (solid red) and excitation (dashed blue) spectra of a solution containing 10 μM $TbCl_3$ and 30 μM A) MB1-1, B) MB1-2, C) MB1-3 and D) MB1-4, at 293 K in 10 mM HEPES buffer pH 7.0, with λ_{em} = 545 nm. Excitation spectrum is corrected for lamp response.

10. Figure S7:

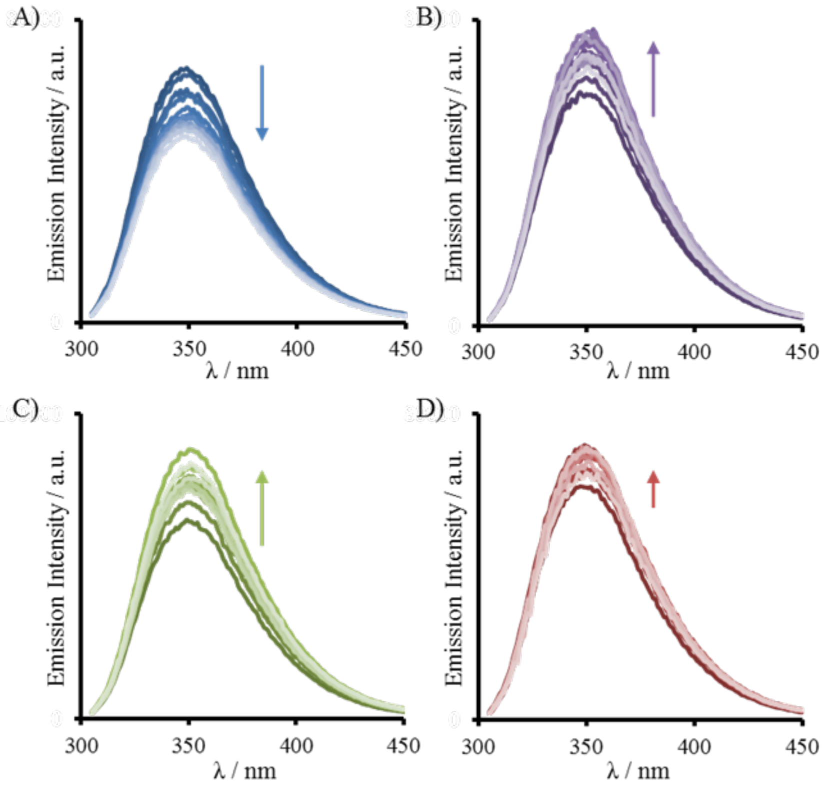

Figure S7. Tryptophan emission spectra upon titration of Tb(III) into 30 μM peptide monomer for A) MB1-1 (blue), B) MB1-2 (purple), C) MB1-3 (green) and D) MB1-4 (red). All samples recorded at 293 K in the presence of 10 mM HEPES buffer pH 7.0. λ_{exc} = 280 nm.

11. Figure S8:

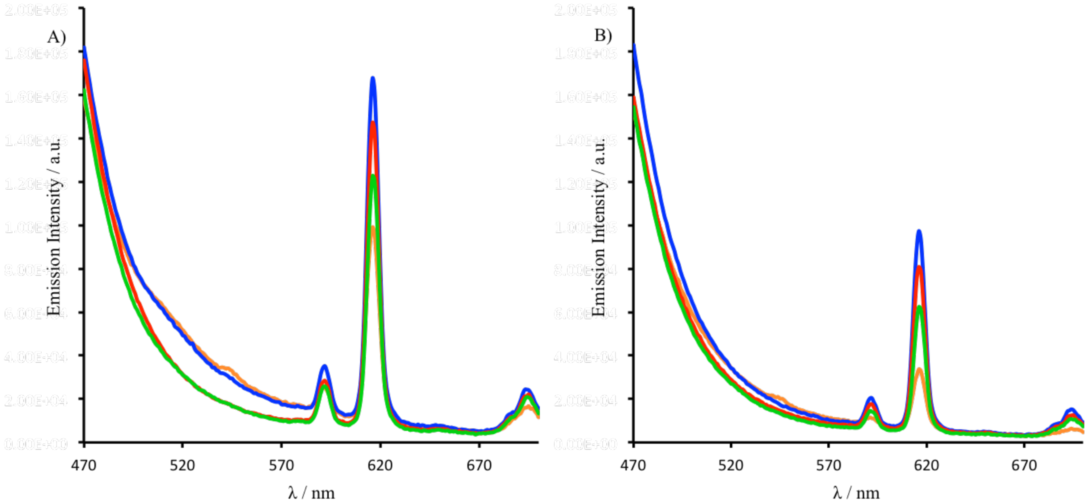

Figure S8. Emission spectra of 30 μM MB1-1 (orange), MB1-2 (blue), MB1-3 (red), MB1-4 (green) monomer peptide recorded in A) D_2O and B) H_2O, in the presence of 10 μM $EuCl_3$, recorded at 293 K in 10 mM HEPES buffer pH 7.0. λ_{exc} = 280 nm.

12. Figure S9:

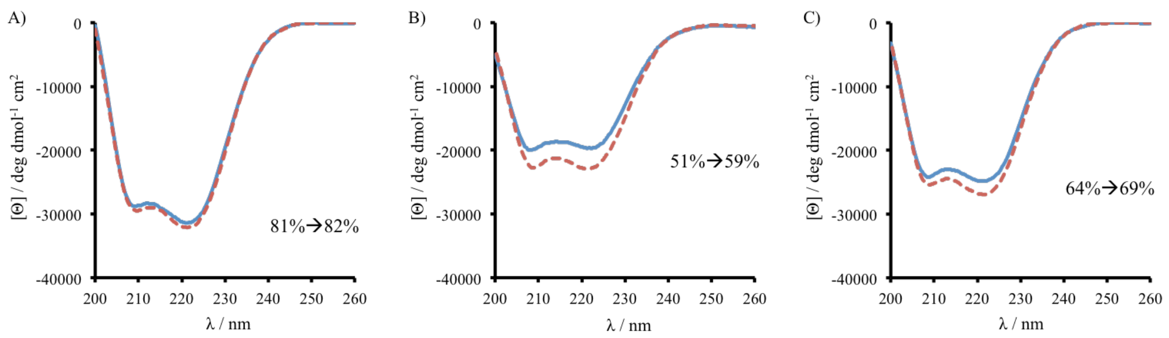

Figure S9. CD spectra of 30 µM A) CS1-1, B) CS1-2 and C) CS1-4 peptide monomer, in the absence (solid blue line) and presence of 10 µM $TbCl_3$ (dashed red line), recorded at 293 K in 5 mM HEPES buffer pH 7.0.

13. Figure S10:

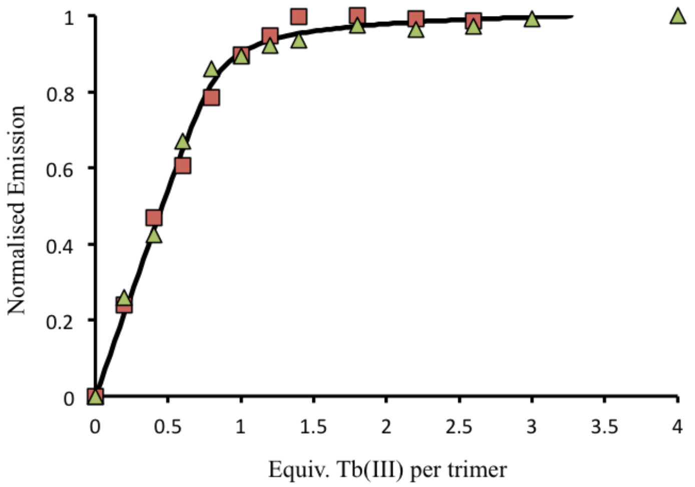

Figure S10. Luminescence titration of $TbCl_3$ into 30 μM CS1-1 peptide monomer in 10 mM HEPES buffer pH 7.0, at 293 K. Data fit to M + 3L → ML_3 model using DynaFit. λ_{exc} = 280 nm.

14. Figure S11:

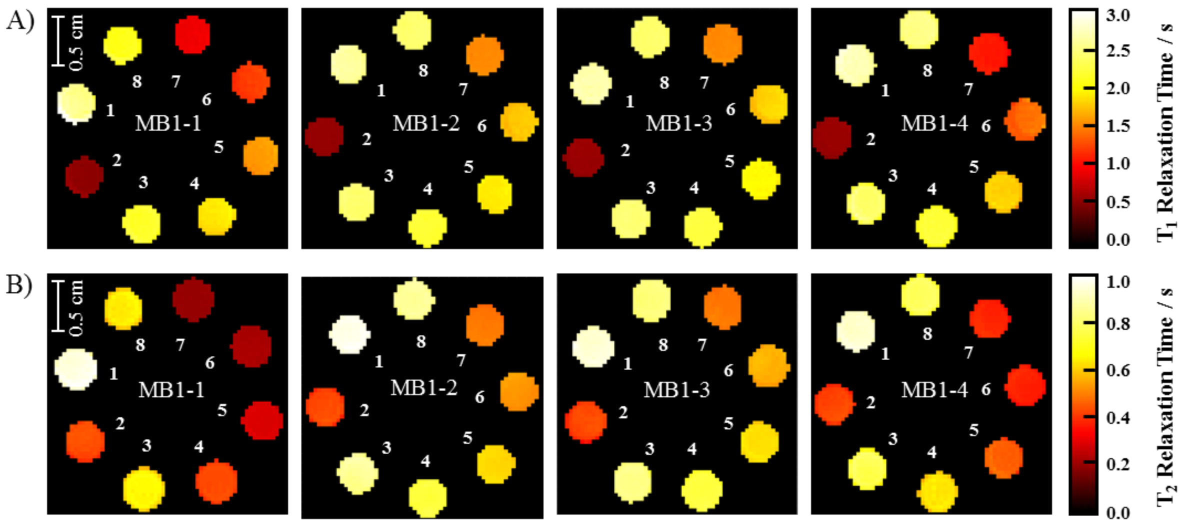

Figure S11. *T*1 (a) and *T*2 (b) NMR relaxation maps for MB1-1 to MB1-4 recorded in 10 mM HEPES buffer pH 7.0, at 293 K. Images recorded using 5 mm NMR tubes containing 10 mM HEPES buffer pH 7.0 (1), 0.1 mM $GdCl_3$ (2), 0.01, 0.02, 0.03, 0.04 and 0.05 mM Gd(III) + 2 equivalences of trimer (3-7), and 0.01 mM Gd(III) + 3 equivalences of trimer (8). Images analysed using PROSPA.

15. Figure S12:

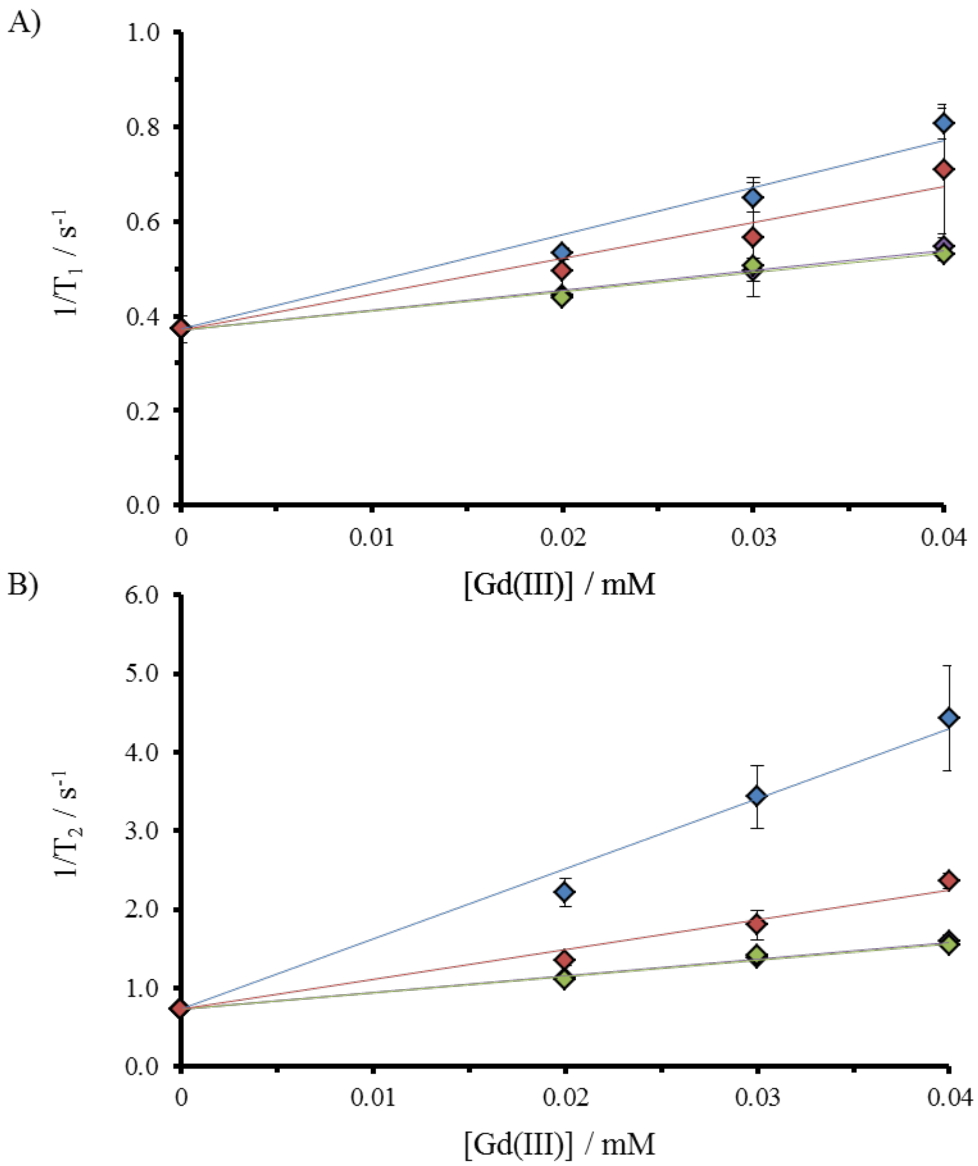

Figure S12. Relaxivity plot showing reciprocal of relaxation time as a function of Gd(III) concentration, for A) *T*1 and B) *T*2 relaxation rates Gd(MB1-1)$_3$ (blue), Gd(MB1-2)$_3$ (purple), Gd(MB1-3)$_3$ (green) and Gd(MB1-4)$_3$ (red). All samples recorded at 293 K in the presence of 10 mM HEPES buffer pH 7.0, with 2 equivalences of peptide trimer to $GdCl_3$. All samples recorded on a 300 MHz NMR spectrometer.

16. Figure S13:

A)

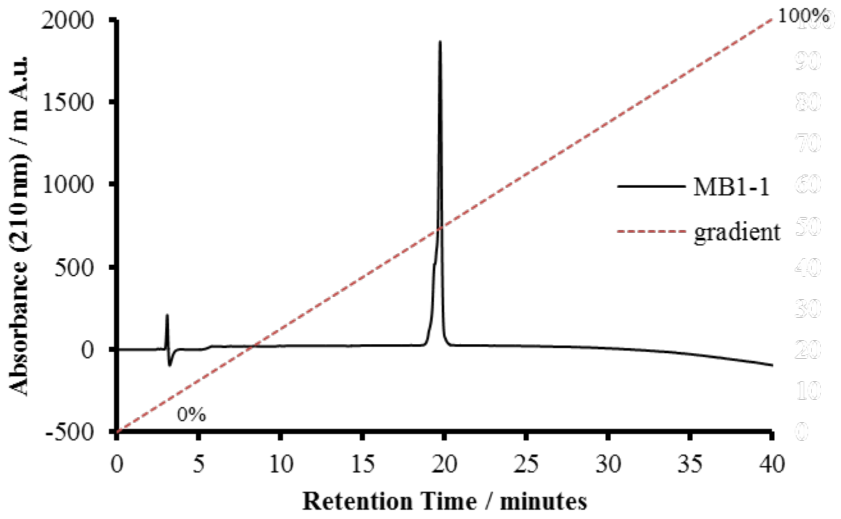

B)

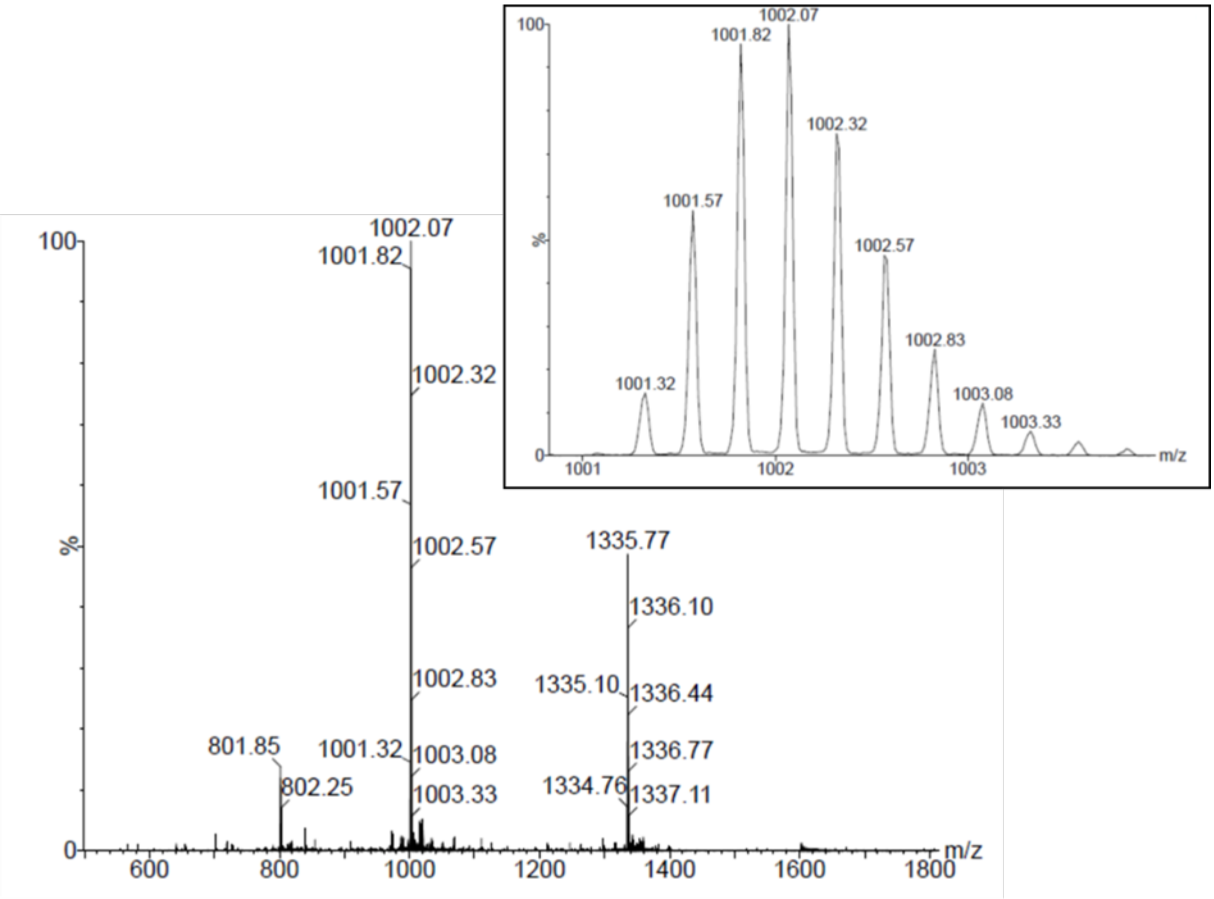

Figure S13. (A) C18-analytical HPLC trace of pure MB1-1 using a linear 0 to 100% MeCN + 0.05% TFA gradient in H_2O + 0.05% TFA over 40 minutes. (B) Electrospray mass spectrum of pure MB1-1 with inset showing isotope distribution from $[M+4H]^{4+}$ peak.

17. Figure S14:

A)

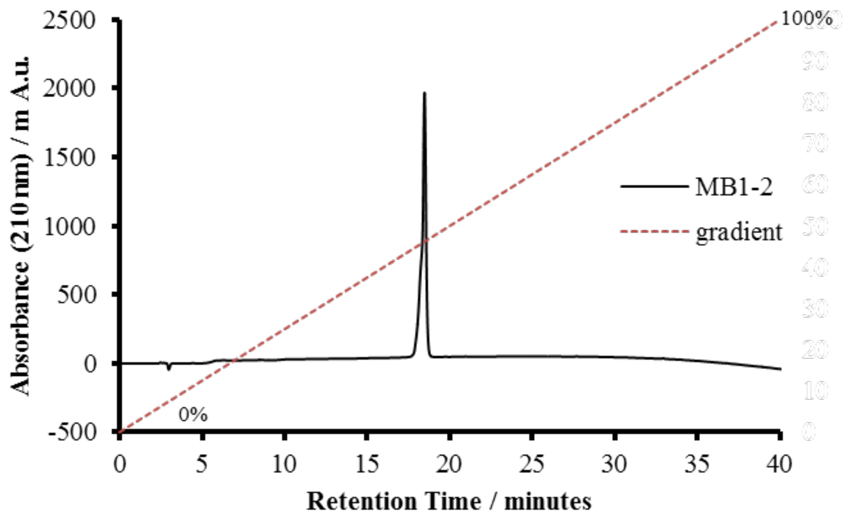

B)

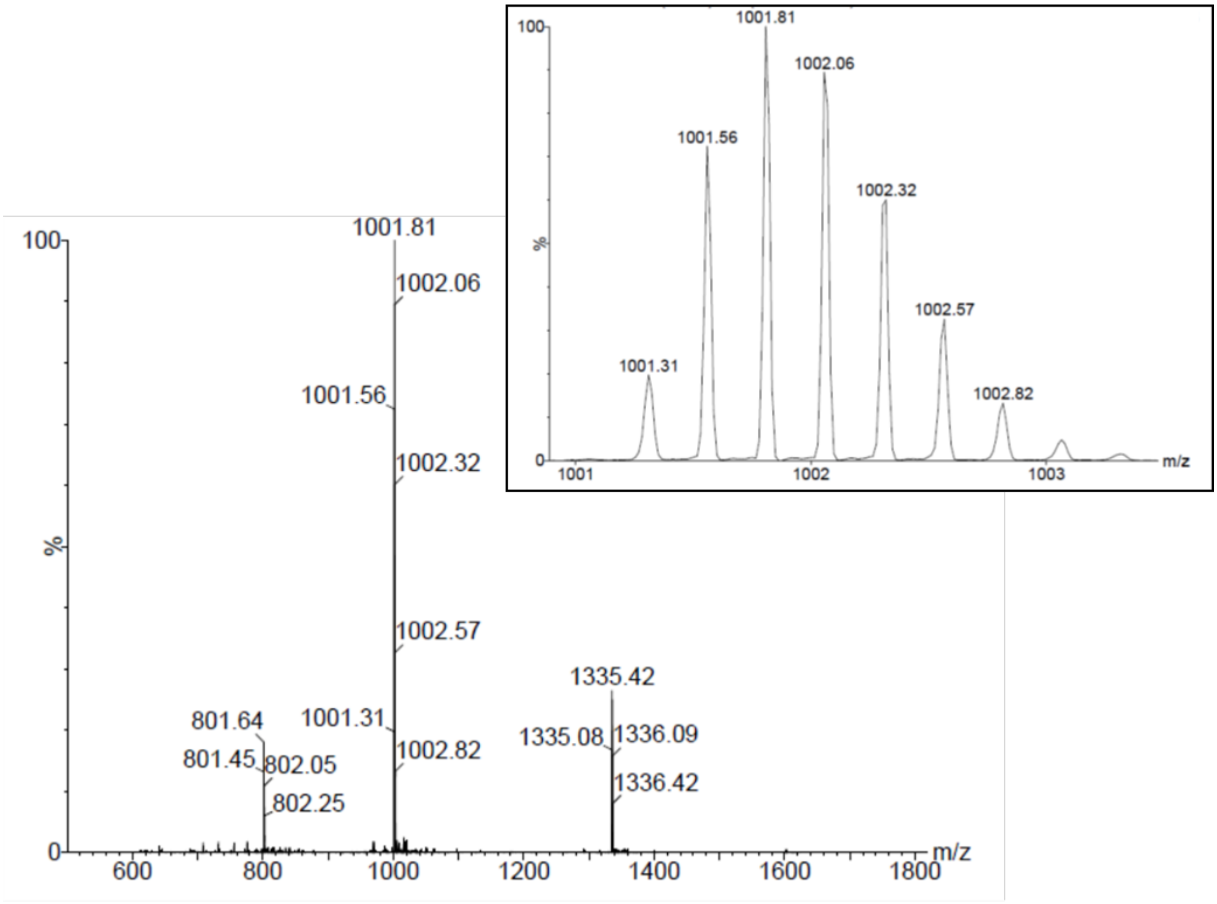

Figure S14. (A) C18-analytical HPLC trace of pure MB1-2 using a linear 0 to 100% MeCN + 0.05% TFA gradient in H_2O + 0.05% TFA over 40 minutes. (B) Electrospray mass spectrum of pure MB1-2 with inset showing isotope distribution from $[M+4H]^{4+}$ peak.

18. Figure S15:

A)

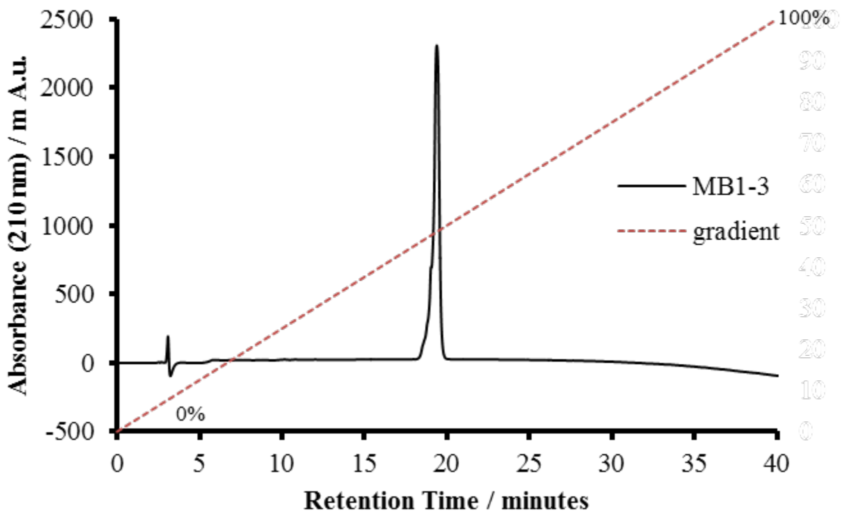

B)

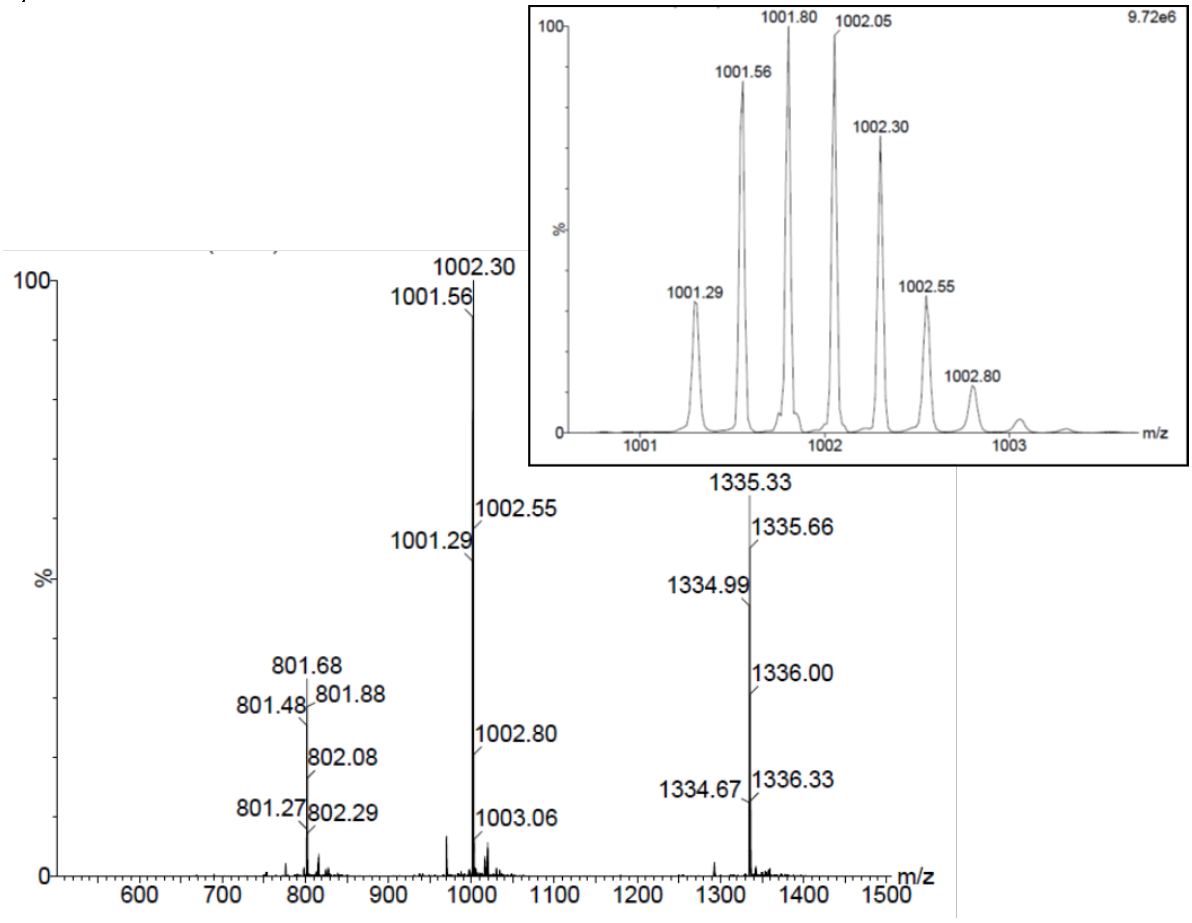

Figure S15. (A) C18-analytical HPLC trace of pure MB1-3 using a linear 0 to 100% MeCN + 0.05% TFA gradient in H_2O + 0.05% TFA over 40 minutes. (B) Electrospray mass spectrum of pure MB1-3 with inset showing isotope distribution from $[M+4H]^{4+}$ peak.

19. Figure S16:

A)

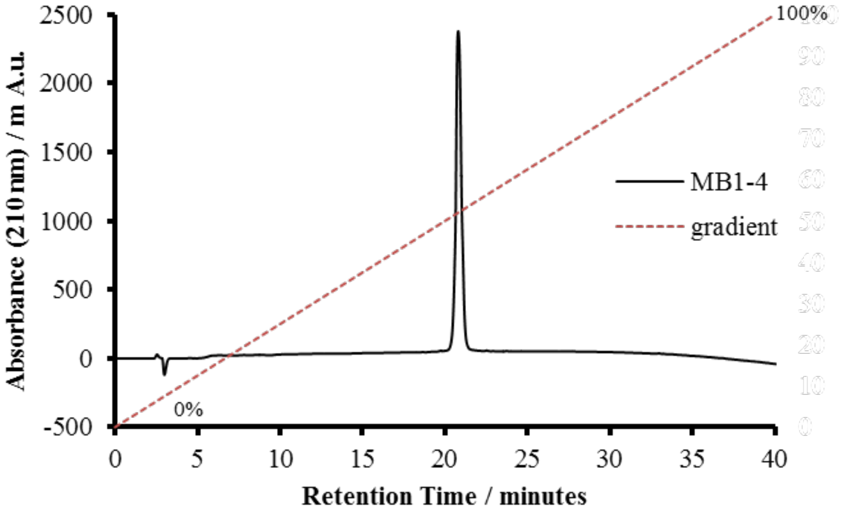

B)

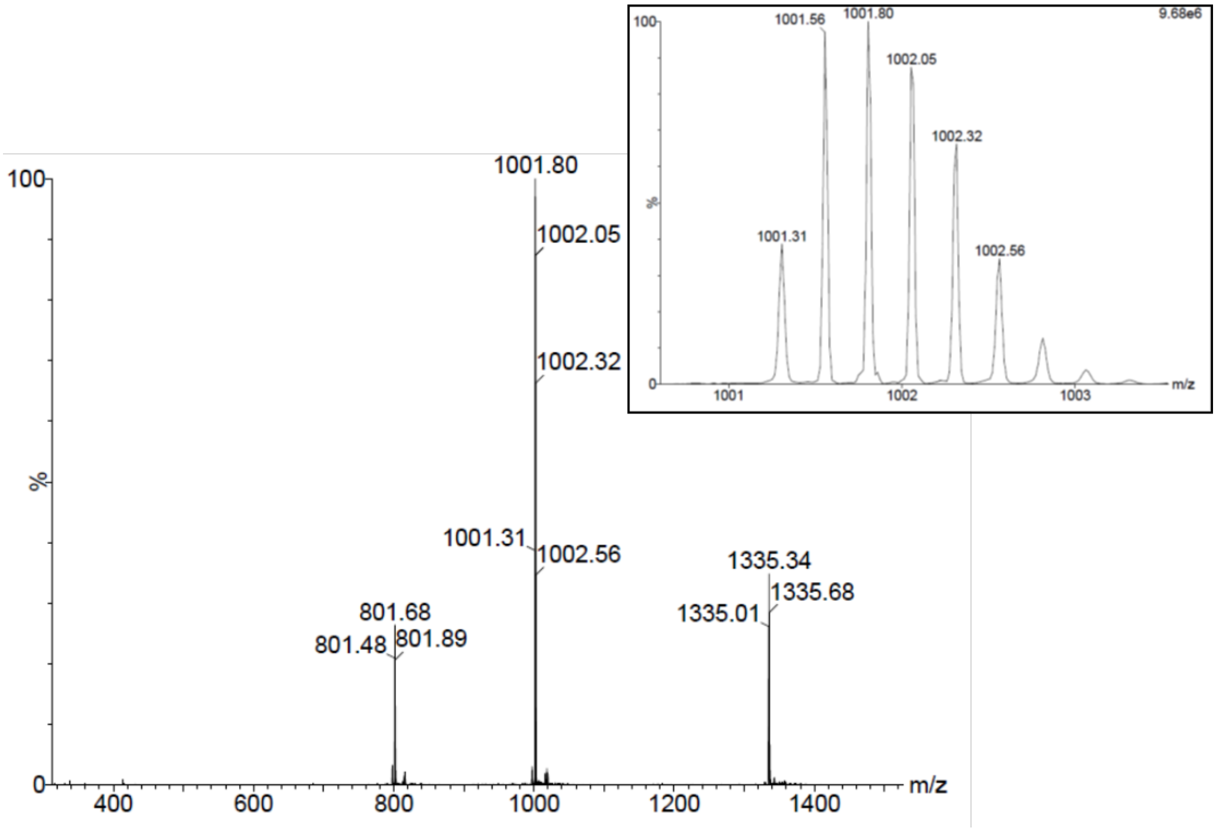

Figure S16. (A) C18-analytical HPLC trace of pure MB1-4 using a linear 0 to 100% MeCN + 0.05% TFA gradient in H_2O + 0.05% TFA over 40 minutes. (B) Electrospray mass spectrum of pure MB1-4 with inset showing isotope distribution from $[M+4H]^{4+}$ peak.

20. Figure S17:

A)

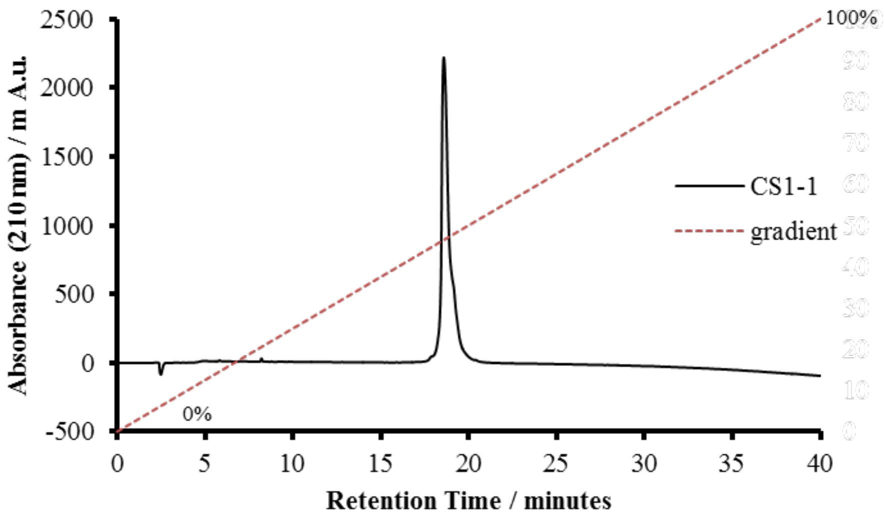

B)

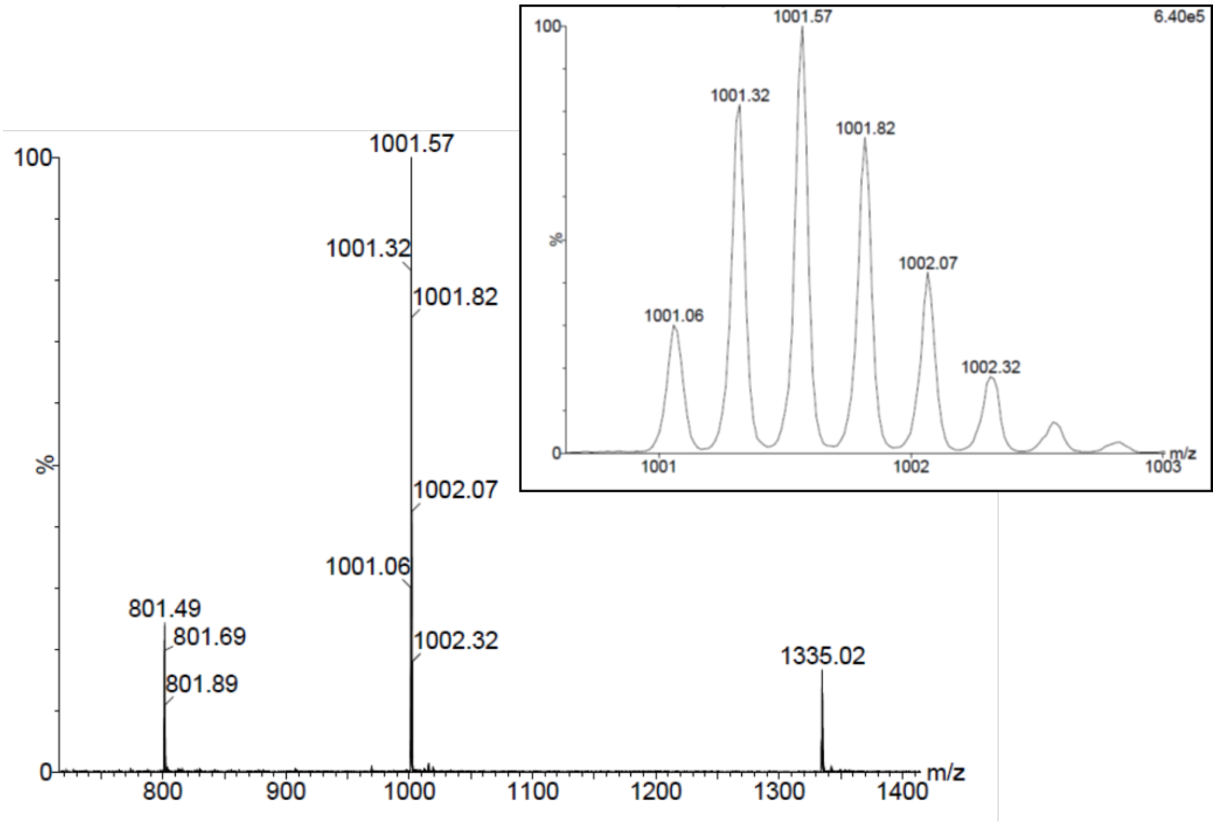

Figure S17. (A) C18-analytical HPLC trace of pure CS1-1 using a linear 0 to 100% MeCN + 0.05% TFA gradient in H_2O + 0.05% TFA over 40 minutes. (B) Electrospray mass spectrum of pure CS1-1 with inset showing isotope distribution from $[M+4H]^{4+}$ peak.

21. Figure S18:

A)

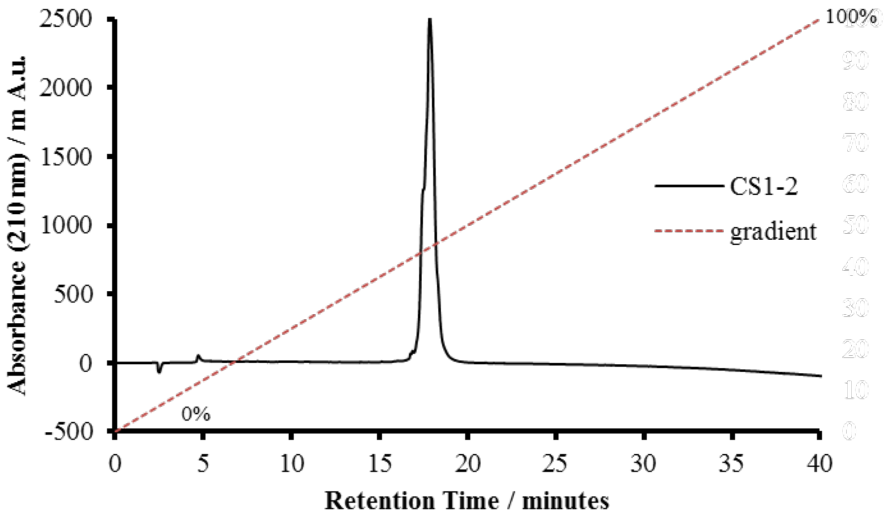

B)

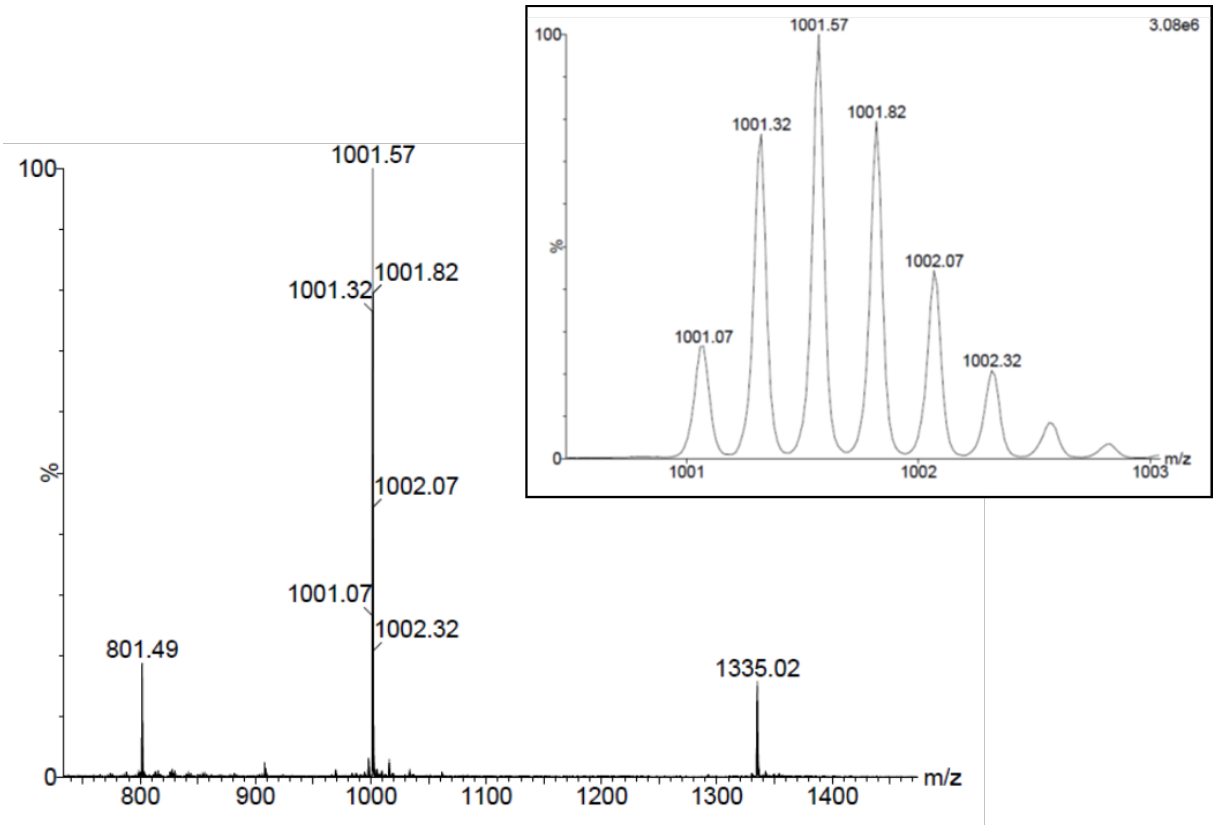

Figure S18. (A) C18-analytical HPLC trace of pure CS1-2 using a linear 0 to 100% MeCN + 0.05% TFA gradient in H_2O + 0.05% TFA over 40 minutes. (B) Electrospray mass spectrum of pure CS1-2 with inset showing isotope distribution from $[M+4H]^{4+}$ peak.

22. Figure S19:

A)

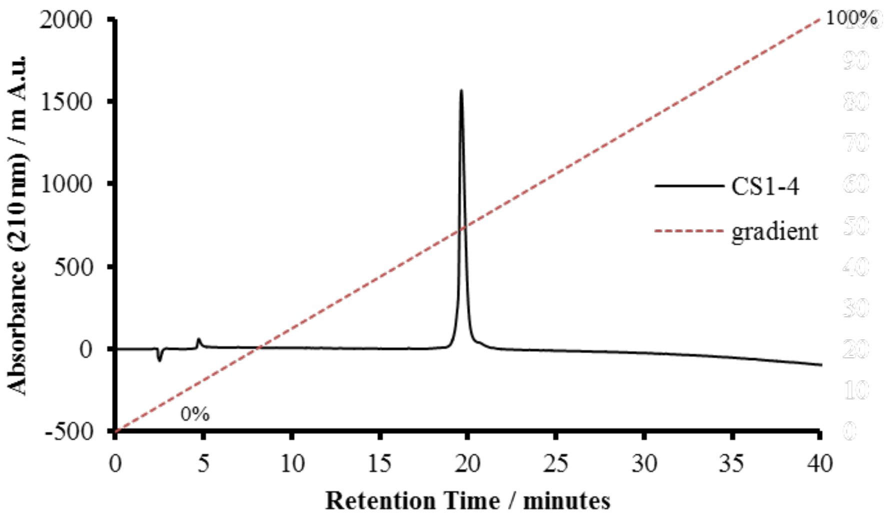

B)

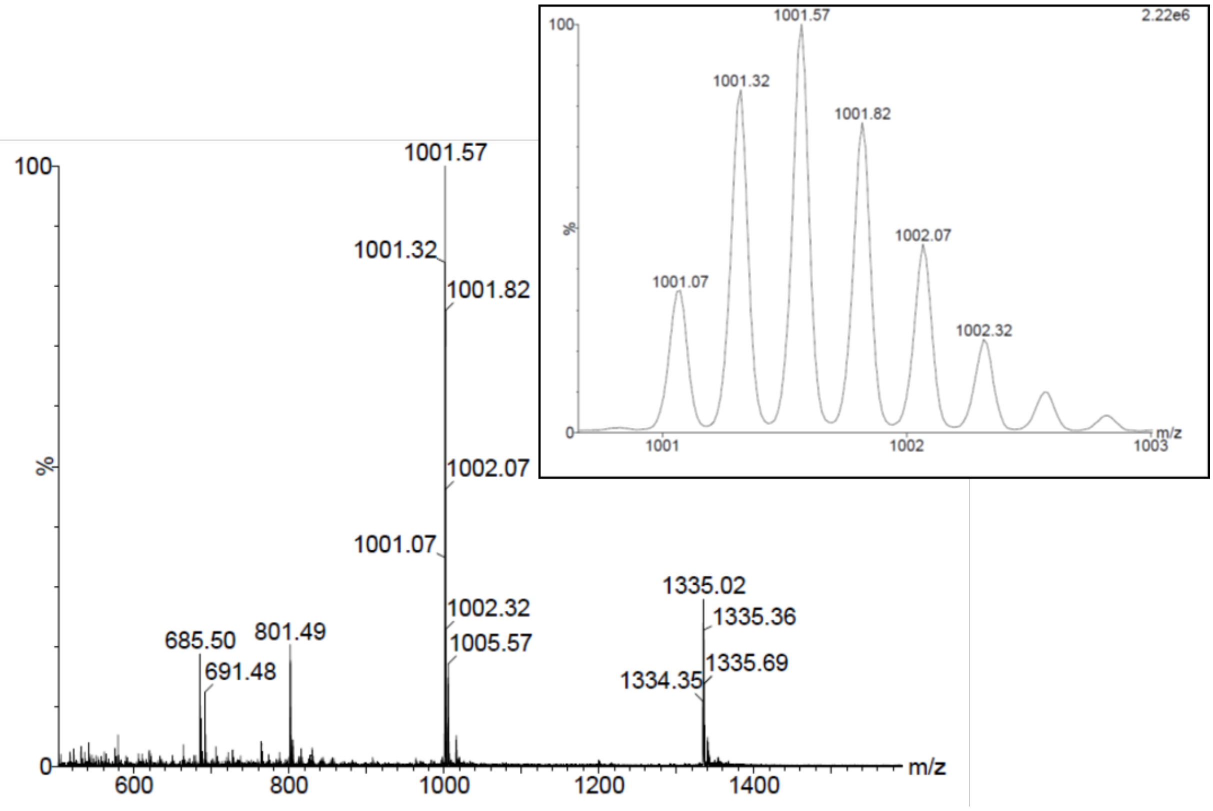

Figure S19. (A) C18-analytical HPLC trace of pure CS1-4 using a linear 0 to 100% MeCN + 0.05% TFA gradient in H_2O + 0.05% TFA over 40 minutes. (B) Electrospray mass spectrum of pure CS1-4 with inset showing isotope distribution from $[M+4H]^{4+}$ peak.

23. References:

1) B. Buer, in *Protein Design: Methods and Applications*, ed. V. Köhler, Humana Press, New York, 2nd edn, 2014, Design, Synthesis, and Study of Fluorinated Proteins, pp 89-116.
2) H. T. Edzes, D. V. Dusschoten and H. van As, *Magn. Reson. Imaging*, 1998, **16**, 185.
3) M. Holz, S. R. Heil, A. Sacco, *Phys. Chem. Chem. Phys.*, 2000, **2**, 4740.
